# New Nanobioceramics Based on Hydroxyapatite for Biomedical Applications: Stability and Properties

**DOI:** 10.3390/nano15030224

**Published:** 2025-01-30

**Authors:** Carmen Steluta Ciobanu, Daniela Predoi, Simona Liliana Iconaru, Catalin Constantin Negrila, Damien Leduc, Liliana Ghegoiu, Coralia Bleotu, Mounsif Ech Cherif El Kettani, Roxana Trusca, Philippe Zelmar, Mihai Valentin Predoi

**Affiliations:** 1 National Institute of Materials Physics, Atomistilor Street, No. 405A, 077125 Magurele, Romania; ciobanucs@gmail.com (C.S.C.); simonaiconaru@gmail.com (S.L.I.); catalin.negrila@infim.ro (C.C.N.); ghegoiuliliana@gmail.com (L.G.); 2 Laboratoire Ondes et Milieux Complexes (LOMC), French National Centre for Scientific Research (CNRS UMR 6294), University Le Havre Normandy, 75 rue Bellot, 76600 Le Havre, France; damien.leduc@univ-lehavre.fr (D.L.); elkettani@univ-lehavre.fr (M.E.C.E.K.); philippe.zelmar@univ-lehavre.fr (P.Z.); 3 Department of Mechanics, University Politehnica of Bucharest, BN 002, 313 Splaiul Independentei, Sector 6, 060042 Bucharest, Romania; 4 Department of Cellular and Molecular Pathology, Stefan S. Nicolau Institute of Virology, Romanian Academy, 030304 Bucharest, Romania; cbleotu@yahoo.com; 5 National Centre for Micro and Nanomaterials, University Politehnica of Bucharest, 060042 Bucharest, Romania; truscaroxana@yahoo.com

**Keywords:** nanobioceramics, hydroxyapatite, zinc, stability, morphology, ultrasound measurements, lyophilization

## Abstract

In this work, we report for the first time the development and complex characterization of new bioceramics based on hydroxyapatite (HAp, Ca_10_(PO_4_)_6_(OH)_2_). On the other hand, the lyophilization process was used for the first time in this research. The samples were obtained by a modified coprecipitation method and were dried by lyophilization (lyophilized hydroxyapatite (HApLF) and lyophilized zinc-doped hydroxyapatite (5ZnHApLF)). Valuable information about the HApLF and 5ZnHApLF stability was obtained through nondestructive ultrasound measurements. The X-ray diffraction (XRD) studies revealed the phase and the effects of the incorporation of Zn ions into the HAp structure. The chemical composition of the samples was evaluated by energy dispersive X-ray analysis (EDS) and X-ray photoelectron spectroscopy (XPS). Information about the functional groups present in the HApLF and 5ZnHApLF was obtained using Fourier Transform Infrared Spectroscopy (FTIR) studies. The morphology of HApLF and 5ZnHApLF pellets was observed by scanning electron microscopy (SEM). The surface topography of HApLF and 5ZnHApLF pellets was studied with the aid of atomic force microscopy (AFM). Details regarding the roughness of the samples were also obtained using AFM topographies and SEM images. A complementary study was also carried out on a larger analysis surface using a Scanning Acoustic Microscope (SAM). The SAM was used for the first time to analyze the surface of HAp and 5ZnHAp pellets. The biological properties of the HApLF and 5ZnHApLF pellets was investigated with the aid of MG63 and human gingival fibroblasts (HGF-1) cell lines. The results of the cell viability assay highlighted that both the HApLF and 5ZnHApLF pellets exhibited good biological activity. Moreover, SEM and AFM studies were conducted in order to emphasize the development of MG63 and HGF-1 cells on the pellet’s surface. Both SEM and AFM images depicted that the pellets’ surface favored the cell attachment and development of MG63 and HGF-1 cells. Furthermore, the antimicrobial properties of the HApLF and 5ZnHApLF were evaluated against *Escherichia coli* ATCC 25922, *Staphylococcus aureus* ATCC 25923, and *Candida albicans* ATCC 10231. The results of the antimicrobial assays highlighted that the 5ZnHApLF exhibited a strong antimicrobial activity against the tested microbial strains. The results of the biological assays suggested that the samples show great potential for being used in the development of novel materials for biomedical applications.

## 1. Introduction

Even though there is an increase in the development of biotechnologies concerning the use of biocompatible materials, the challenge remains to find new solutions for the increasingly diverse pathologies that society faces today. Hydroxyapatite (HAp, Ca_10_(PO_4_)_6_(OH)_2_) is a biocompatible ceramic material with multiple similarities to bone tissue [1], which makes it one of the few ceramic materials capable of forming grafts with natural tissue through the biochemical bonds it establishes with bone tissue [2,3]. Its biocompatibility, osteoconductivity, and ability to facilitate bone regeneration have made HAp a widely studied material with potential applications in the biomedical area. However, HAp requires certain improvements regarding mechanical strength and antimicrobial activity. For this purpose, the technique of doping with various transition metal ions has been employed. One of the studied dopants reported in the literature is represented by Zn ions (Zn^2+^). Doping HAp with zinc involves the partial substitution of Ca^2+^ ions in the HAp crystalline network with zinc ions (Zn^2+^), thereby improving the biological and physicochemical properties of the resulting composite.

Zinc is one of the essential trace elements with an important role in the normal development and growth of the skeletal system. Approximately 30% of the total Zn in the body is stored in bone tissue. A Zn deficiency negatively impacts skeletal development and is associated with the pathogenesis of osteoporosis [4,5]. It is well-acknowledged that Zn can stimulate bone formation and enhance the osteogenic response in osteoblasts by increasing cell proliferation, gene expression related to osteogenesis, and extracellular matrix synthesis [6].

Zinc has gathered considerable attention from researchers around the world due to its ability to improve cell viability, proliferation, and adhesion, as well as due to its antibacterial characteristics, which are advantageous in minimizing infections in bone tissue [7].

Regarding the antimicrobial activity of zinc, previous studies have shown that Zn exhibits greater antibacterial efficacy against *Staphylococcus aureus* compared to *E. coli* [8]. Furthermore, Zn-doped HAp coatings are more effective against *S. aureus* than against *Bacillus cereus* [9]. Antimicrobial activity, stimulation of bone regeneration, enhancement of mechanical properties, and control over biodegradability (zinc can regulate the degradation rate of HAp, enabling better integration into biological tissues) are among the benefits of using Zn-doped HAp.

Lyophilization, or freeze-drying, is a process used to remove water from a material through sublimation. For Zn-doped HAp, lyophilization was chosen as the drying method to obtain a porous, uniform powder with optimized properties for biomedical applications. The freeze-drying technique was successfully used by Zhang, X. and colleagues, who created complex fibers using polymers such as chitosan [10]. The applications of structures obtained via lyophilization cover various fields. Niziołek, K. et al. investigated the effect of the drying technique on the porosity and transport capacity of active substances in HAp synthesized in the laboratory [11].

This study aims to create a composite that demonstrates improvements in HAp properties (via Zn doping), through lyophilization. The resulting composite may be of interest in orthopedic and dental implants, as Zn-doped HAp could accelerate implant integration into bone tissue and provide antimicrobial activity against various microbial strains, according to previous studies reported in the literature [1,2,3,4,5,6,7,8,9,10,11].

Obtaining compact and homogeneous materials from powders, following the pressing process, has generated great interest for a long time [12,13,14]. In order to achieve a ceramic material with advanced properties for the production of compact biocompatible materials or coatings, this study aims to obtain for the first time HAp and zinc-doped HAp powders (x_Zn_ = 0.05) obtained after lyophilizing the precipitates resulting from the synthesis using an adapted co-precipitation method. This research study introduces a novel approach by employing lyophilization as a drying method for the first time in the production of HAp and 5ZnHAp powders, resulting in enhanced physicochemical and biological properties. Thus, the dispersion of nanometric particles of HApLF and 5ZnHApLF powder (in water), based on their stability, can lead to the formation of a homogeneous and dense consolidated material with minimal defects.

## 2. Materials and Methods

### 2.1. Synthesis of Hydroxyapatite and Zinc-Doped Hydroxyapatite

For the development of hydroxyapatite (HAp) and zinc-doped hydroxyapatite (ZnHAp), the following main precursors were used: ammonium hydrogen phosphate ((NH_4_)_2_HPO_4_; Wako Pure Chemical Industries Ltd., Richmond, VA, USA), calcium nitrate (Ca(NO_3_)_2_·4H_2_O, 99% purity, Aldrich, St. Louis, MO, USA), and zinc nitrate (Zn(NO_3_)_6_·6H_2_O (Alpha Aesare, Langen, Germany, 99.99% purity).

Hydroxyapatite (HAp) and zinc-doped hydroxyapatite (HAp, 5ZnHAp, x_Zn_ = 0; 0.05, Ca_10−x_ Zn_x_(PO_4_)_6_(OH)_2_) were obtained with the aid of an modified coprecipitation method, in agreement with our previous work [15]. HAp and 5ZnHAp samples were prepared in air at 80 °C using aqueous solutions and maintaining the [Ca + Zn]/P ratio at 1.67. In the next section, only the development of 5ZnHAp samples [15] will be described. Thus, a first solution containing appropriate quantities of Ca(NO_3_)_2_·4H_2_O and Zn(NO_3_)_2_·6H_2_O dissolved in deionized water was obtained. A second solution containing appropriate quantities of (NH_4_)_2_HPO_4_ dissolved in deionized water was also achieved. The next step was represented by adding the second solution into the first solution under vigorous and continuous stirring for 72 h at 80 °C [15]. During the fabrication process, the pH of the resulting suspension was adjusted to 10. Then, the resulting white precipitate was washed several times with deionized water and centrifuged [15]. Finally, the obtained HAp and ZnHAp precipitates were dried by lyophilization. The obtained specimens shall be further referred to as HApLF and 5ZnHApLF, respectively.

### 2.2. Characterization Techniques

In order to evaluate the phase and the effects of the incorporation of Zn ions into the HAp structure, a Bruker D8 Advance diffractometer (Bruker, Karlsruhe, Germany) equipped with a LynxEye™ 1D high-efficiency one-dimensional linear detector was used. The radiation used was CuKα (λ = 1.5418 Å). Data collection was carried out in the 2θ range of 20–80°, with a step size of 0.02° and a time of 5 s per step. The average crystallite size of the HApLF and 5ZnHApLF samples was calculated using the Scherrer formula [16].

$$D_{hkl}=\frac{K\lambda }{\beta_{hkl}cos\theta_{hkl}}$$

where D represents average crystallite sizes, K is Scherrer constant (0.94), β is the Full Width at Half Maximum (FWHM), (h, k, l) represent the Miller indices, θ is the Bragg diffraction angle, and λ is the wavelength of the monochromatic X-ray beam (1.54 Å).

The lattice parameters “a” and “c” were determined taking into account the positions of the (hkl) peaks of the analyzed samples from the XRD patterns, in accordance with the following formula [17,18]:

$$\frac{1}{d^{2}}=\frac{4}{3}\left[\frac{h^{2}+k^{2}+hk}{a^{2}}\right]+\frac{l^{2}}{c^{2}}$$

where d is the distance between two adjacent planes (hkl).

The unit cell volume was calculated using the following formula:

$$V=\frac{3\sqrt{3}}{2}a^{2}c$$

The degree of crystallinity, representing the proportion of the crystalline phase within the examined volume, was determined using the following relation [19]:

$$\beta_{002}\cdot {\left(X_{C}\right)}^{1/3}=K$$

where β is the Full Width at Half Maximum (FWHM) of the (002) reflection peak, and K is a constant.

The stability of the HApLF and 5ZnHApLF lyophilized powders suspensions was assessed using non-destructive ultrasound (US) measurements. The double-distilled water was used as reference. Our previous study [20] described the protocol and instrument used in US studies. A quantity of 100 mL of HApLF and 5ZnHApLF lyophilized powders suspensions was continuously stirred at room temperature for 15 min, at 800 rot/min, in order to obtain a good homogeneity of the solid particles. The suspension was poured in a special transparent cubical container equipped with two coaxial ultrasonic transducers which are spaced 16 mm apart. The axis of the transducers is positioned at the mid-height of the container box. Immediately after stopping the stirring machine, the acquisition of the 1000 ultrasonic signals began, by recording one signal every 5 s on the digital oscilloscope. Each recorded signal was an average of 32 signals on the oscilloscope, reducing the experimental noise.

The Fourier Transform infrared spectra for the HApLF and 5ZnHApLF samples were obtained using a Perkin Elmer SP 100 spectrometer (Waltham, MA, USA), which was equipped with an Attenuated Total Reflection (ATR) accessory. The experimental data were collected within the spectral range of 450–2000 cm^−1^ with a resolution of 4 cm^−1^ [21]. The samples were analyzed directly on the ATR accessory without any prior preparation. Additional information about the fine structure of the absorption spectra for HApLF and 5ZnHApLF was achieved through a peak fitting analysis, which involved baseline correction and second-order derivative calculations to identify peak positions. The peak fitting analysis steps were previously described in detail [21].

The charge compensation was conducted by a flood gun of the Specs FG15/40 type. The acquirement was made with a Pass Energy of 20 eV for the individual spectra and 50 eV for the extended spectrum. CasaXPS 2.3.14 software (utilizing the Shirley background type) was used for data analysis [22]. Furthermore, the XPS tables were referenced [23,24]. All binding energy (BE) values presented in this research were charge-corrected to C 1 s at 284.8 eV.

The surface morphology of the HApLF and 5ZnHApLF pellets was examined using atomic force microscopy (AFM) with a NT-MDT NTEGRA Probe NanoLaboratory system (NT-MDT, Moscow, Russia). The studies were performed in non-contact mode at room temperature and in atmospheric conditions. The topographies were captured with a silicon NT-MDT NSG01 cantilever, having a 35 nm gold-coated tetrahedral tip. The 2D AFM micrographs were recorded on a surface area of 10 × 10 μm^2^. The surface topographies and their 3D representations were analyzed using Gwyddion 2.55 software [25]. Additionally, the surface roughness was assessed by determining the root mean square roughness (R_RMS_) from the 2D AFM topography.

Information regarding the morphology of HApLF and 5ZnHApLF pellet surfaces was obtained using a scanning electron microscope (FEI Quanta Inspect F, FEI Company, Hillsboro, OR, USA). In order to obtain information about the chemical composition of the samples, the energy dispersive X-ray analysis (EDS) was employed.

Preliminary data about the Vickers hardness of the studied samples were obtained with the aid of the Microtech MX3 system (Microtech, Fletcher, NC, USA), which includes the hardness tester model MMT-X3 (Matsuzawa Co., Ltd., Niigata, Japan). For this study, the Vickers hardness was measured at a load of 200 g (with a dwell time of 10 s), and each sample was indented three times in order to obtain the statistical average of hardness values [26].

The surface morphology of the pellets was also examined by acoustic microscopy using a PVA TePla SAM301 device (PVA TePla GmbH, Wettenberg, Germany). Measurements were taken in immersion at 21 °C in pulse-echo mode using a PT-100 transducer manufactured by PVA-Tepla, with a center frequency of 83 MHz. Surface imaging (C-Scan) was carried out on a 3 × 3 mm^2^ surface with an X-scan time window of 20 ns. The evolution of the maximum of the signal reflected from the surface was plotted using Winsam 8 software (microscope proprietary software) and then displayed in 3D using Gwyddion 2.55 software.

### 2.3. In Vitro Biological Assays

The biological properties of HApLF and 5ZnHApLF were investigated using MG63 and the human gingival fibroblast (HGF-1) cell line, employing an experimental design previously described in detail by Iconaru, S.L. et al. [27]. The cells were grown using Dulbecco’s Modified Eagle’s Medium (DMEM), supplemented with fetal bovine serum (FBS), penicillin–streptomycin, and L-glutamine. The cultures were maintained at a temperature of 37 °C in a humidified atmosphere having 5% CO_2_. The HApLF and 5ZnHApLF pellets were placed in 24-well plates, ensuring that each well contained one pellet, and the MG63 cells were seeded at a density of 1 × 105 cells/well. A quantitative assay using MTT (3-(4,5-dimethylthiazol-2-yl)-2,5-diphenyl tetrazolium bromide) was used to determine the cell viability after 24, 48, and 72 h of incubation. For this purpose, after the incubation periods, the MG63 cells were washed using phosphate-buffered saline (PBS) and incubated with 1 mg/mL MTT solution for 3 h in the dark. Afterwards, the cell viability was quantified through the optical density of the medium at 595 nm using a TECAN spectrophotometer (TECAN, Männedorf, Switzerland). The percentage of viable MG63 cells was determined in comparison to a control sample, which exhibited 100% viability. Furthermore, after 72 h of incubation, the adhered cells were fixed on the pellets’ surface with the aid of a 2.5% glutaraldehyde solution, dehydrated using ethanol, and air-dried. The as-prepared samples were examined by SEM and AFM in order to investigate the cells adherence and development on the pellets’ surface, as well as interaction between the cells and the pellet surface.

The antimicrobial activity of the samples was determined with the aid of *Escherichia coli* ATCC 25922, *Staphylococcus aureus* ATCC 25923 and *Candida albicans* ATCC 10231 microbial strains. The antimicrobial properties were evaluated using an adapted standard method [28] to perform the quantitative assays [29]. The antimicrobial quantitative assays were conducted by exposing the samples to 1.5 mL of microbial suspensions containing 5 × 10^6^ CFU/mL in phosphate-buffered saline (PBS). The microbial suspensions were subsequently collected at specific time intervals (24, 48, and 72 h) and incubated for 24 h on LB agar medium. A free microbial culture was used as the positive control (C+). The colony-forming units per milliliter (CFU/mL) were quantified. All the experiments were performed in triplicate, and the results were presented graphically as mean ± standard deviation (SD).

## 3. Results

**Figure 1:**
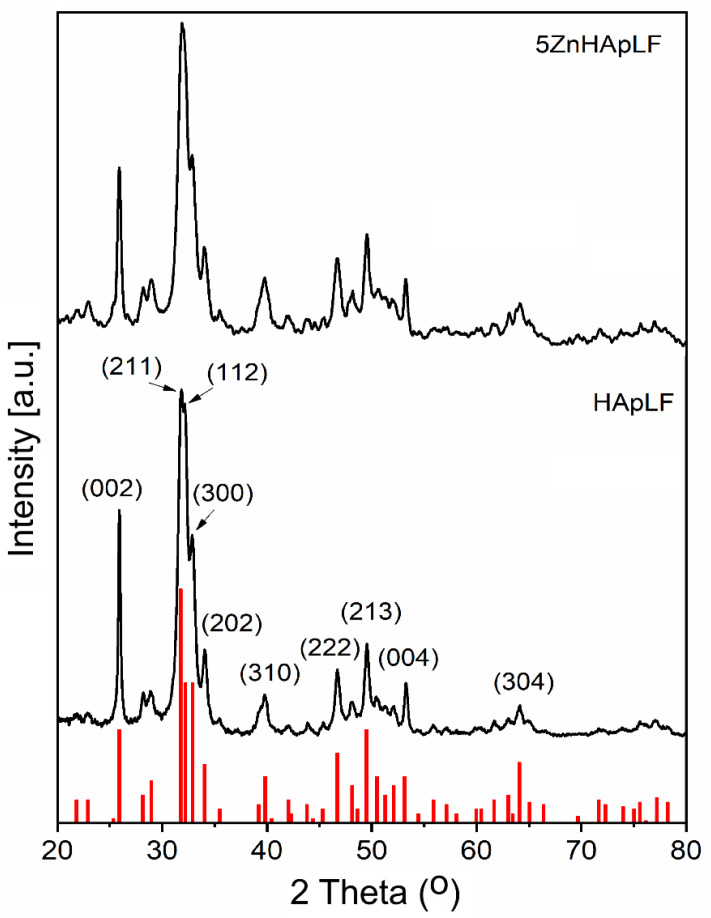
XRD spectra of HApLF and the 5ZnHApLF composite. The red lines represent the JCPDS file no. 09-432 of pure hexagonal hydroxyapatite.

The XRD patterns of the HApLF and 5ZnHApLF samples are presented in Figure 1. As can be seen in Figure 1, for a good evaluation of the phase and the effects of the incorporation of Zn ions into the apatite structure, a comparison was made with the JCPDS file no. 09-432. The XRD patterns of the HApLF and 5ZnHApLF samples showed peaks specific to pure hexagonal HAp (P63m). Replacing calcium with zinc ions led to a decrease in intensity and a slight broadening of the peaks in the case of the 5ZnHApLF sample. The concentration of zinc incorporated into the HAp structure being low (5ZnHApLF sample), no influence was observed in the decrease in crystallinity compared to the HApLF sample. In agreement with LeGeros [30], the incorporation of large amounts of Zn^2+^ into the HAp structure can lead to defects in the crystal structure. As can be seen, there were slight changes in the peak positions for the 5ZnHApLF powder compared to HApLF.

In accordance with Gamal et al. [31], it is assumed that the higher structural strain and smaller particle size of the 5ZnHAp powder could be responsible for this behavior. In order to highlight the lattice defects as a result of the substitution of Ca^2+^ ions with Zn^2+^ in the HAp structure, both the unit cell parameters (“a” and “c”) and the unit cell volume (V) were calculated (Table 1). The values obtained were compared to the values of pure HAp (JCPDS no. 09–432). It was observed that the lattice parameter “a” increased in the case of the 5ZnHApLF sample, while the value of this parameter for the HApLF sample were slightly lower (9.415 A compared to 9.418 A). The value of the lattice parameter “c” was lower in the case of the 5ZnHApLF sample compared to the HApL sample and to pure HAp. Moreover, in the case of the 5ZnHAp sample, the value of the lattice parameter “c” decreased drastically (Table 1). In their study Fumiaki et al. [32] also reported an increase in the lattice parameter “a” and a decrease in the lattice parameter “c” when calcium ions were replaced by zinc ions. The calculated unit cell volume for both samples was smaller than that of pure HAp (JCPDS no. 09–432). The calculated unit cell volume for the 5ZnHAp sample was larger than that of the HApLF sample (Table 1). In agreement with previous studies [33,34], we can say that the defects that appeared are due to the replacement of calcium ions with zinc ions. The crystal size of the HApLF and 5ZnHApLF powders was calculated using the Scherrer formula [35] on (002), (211), (112), (300), and (302) reflections. It was observed that the crystallite size decreases in the case of the 5ZnHApLF sample (16.92 ± 2 nm) compared to that of the HapLF sample (20.66 ± 2 nm). The ZnHApLf sample exhibited lower and broader reflections compared to HApLF, indicating a lower crystallinity of the HAp phase. The crystallinity values estimated from X-ray diffraction data for both samples are presented in Table 1.

In order to obtain a ceramic material that can be used in different medical areas, the stability of HAp and 5ZnHAp powders resulting from lyophilization of the precipitate obtained from the synthesis was studied. Furthermore, the dispersion behavior of these HAp and 5ZnHAp powders resulting from lyophilization was analyzed by ultrasound measurements.

**Figure 2:**
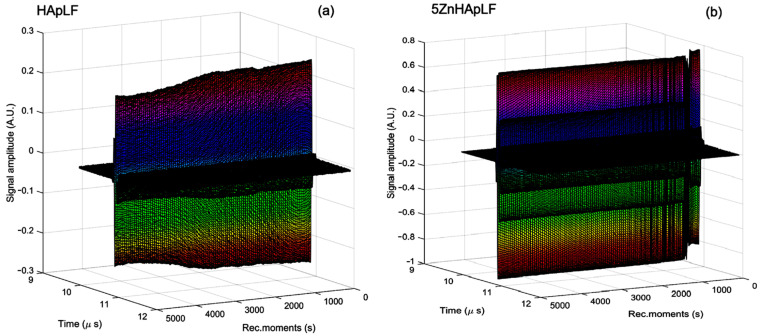
Time evolution of the recorded signals for HApLF (**a**) and 5ZnHApLF (**b**) samples, from left to right, over a duration of 5000 s.

A superposition of the 1000 recorded signals for the HApLF and 5ZnhApLF samples is shown in Figure 2.

**Figure 3:**
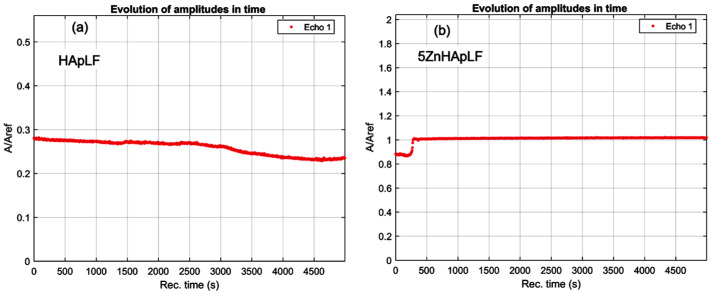
Recorded signals amplitudes during the experiment for the HApLF (**a**) and 5ZnHApLF (**b**) samples.

All these signals, representing waterflow, are plotted from right to left, covering 5000 s of process evolution. The sedimentation process is rapid, with variations of the overall signal amplitude lasting 250 s for both samples. This evolution is detailed in Figure 3a, showing a continuous and irregular reduction in amplitude for sample HApLF. For the 5ZnHApLF sample, this evolution is detailed in Figure 3b, showing a slow initial reduction in amplitude. After 250 s, the sedimentation free surface passes in front of the transducers, after which the suspension becomes stable.

**Figure 4:**
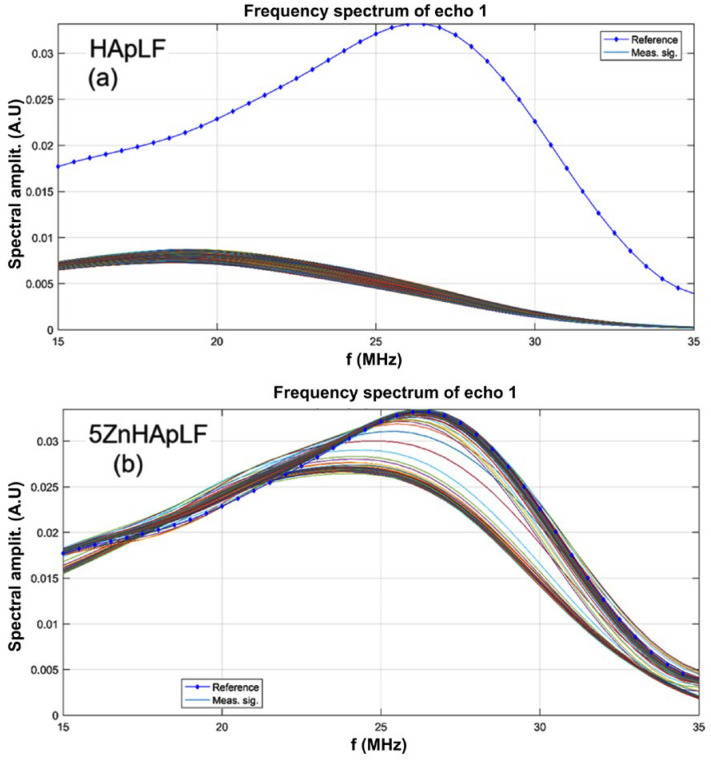
Spectral amplitudes of all recorded signals for the HApLF (**a**) and 5ZnHApLF (**b**) samples.

The evolution of the frequency spectrums for each of the 1000 signals of the HApLF and 5ZnHApLF samples is shown in Figure 4. The spectrum of the reference liquid (double distilled water, in dotted blue line) is also plotted for comparison. In Figure 4a (HApLF sample), the compactness of the 1000 curves is remarkable, indicating a stable composition of the suspension, but showing a strong attenuation of this sample. In the case of the 5ZnHApFL sample (Figure 4b), it can be remarked that the spectra are grouped in two families: one with lower amplitudes, representing the short initial rapid evolution, and the other representing the stable suspension, but which is very close to the reference liquid. The peaks of the short evolution are located at 24 MHz, below the 26.2 MHz peak of the reference liquid.

**Figure 5:**
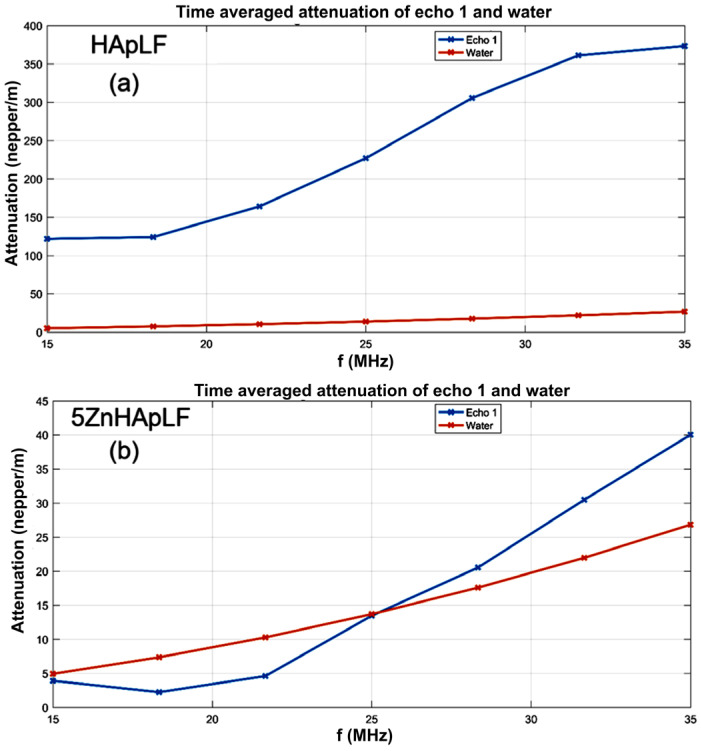
Time averaged attenuation for the investigated frequency range for the HApLF (**a**) and 5ZnHApLF (**b**) samples.

**Figure 6:**
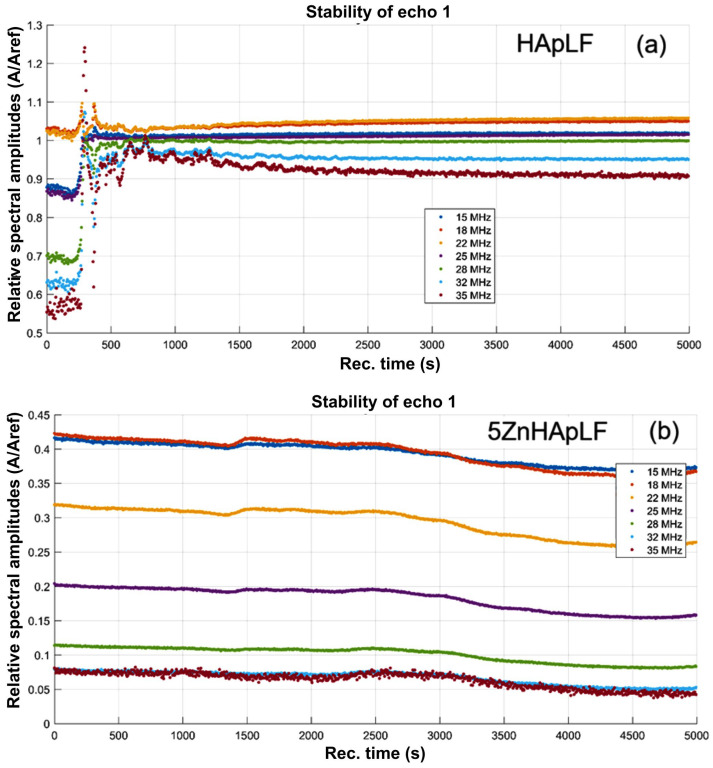
Relative spectral amplitudes vs. time of the HApLF (**a**) and 5ZnHApLF (**b**) samples.

The temporal evolution of signals frequency spectra, which is related to the properties of the suspension in front of the transducers, bring more insight into the attenuation process. The time-averaged attenuation plots for HApLF and 5Zn HApLF are shown in Figure 5. The attenuation of the HAp sample (Figure 4a) is very important, ranging from 120 nepper/m at lower frequencies up to 370 nepper/m at higher frequencies. For the 5ZnHApLF (Figure 5b), it should be emphasized that for the most duration, the suspension is almost similar to the reference liquid. However, the initial rapid evolution is manifesting by an overall lower attenuation associated with the lower frequencies and for frequencies above 25 MHz. On the other hand, the attenuation increases with increasing frequency, but at a higher rate than in the reference liquid (Figure 5b). Another characteristic of the HApLF and 5ZnHApLF suspensions is the spectral stability, representing the amplitude of the frequency component of each spectrum, as function of time (Figure 6). The sample HApLF shows a similar attenuation for frequencies in the 15–18 MHz range and also in the 32–35 MHz range, but with large differences between the relative amplitudes of these two ranges (Figure 6a). The HApLF sample can be considered relatively unstable, the stability parameter being $S=\frac{\bar{dA}}{Adt}$ = 3.05·10^−5^ s^−1^, in which *A* is the signal amplitude, with the bar above indicating time averaging.

Some remarkable features of the 5ZnHApLF sample (Figure 6b) can be attributed to its properties. During the initial 250 s, the rapid sedimentation produces a slow reduction in spectral amplitudes, but considerably dependent on frequency. For the frequency range of 18–22 MHz, the amplitude is larger than in the reference liquid, but at 15 MHz and 25 MHz, the signal is more attenuated with amplitude ratios below 0.88.

The higher frequencies composing the signal are even more attenuated. After the transitory period lasting up to about 1000 s after the start of the test, the spectral amplitudes tend towards stable values, leaving the same pattern of amplitudes, with two being above the amplitudes in the reference liquid. This behavior proves the existence of a resonant frequency of the suspension, between 18 and 22 MHz. The 5ZnHApLF sample is very unstable in its original state, many nanoparticles settling within about 250 s. After this rapid evolution, the stability parameter becomes relatively small, indicating a relatively good stability: $S=\frac{\bar{dA}}{Adt}$ = 1.44·10^−4^ s^−1^, in which *A* is the signal amplitude, with the bar above indicating time averaging.

Ultrasound measurements showed that HApLF powders are less stable than 5ZnHApLF powders. This behavior could be explained by the substitution of calcium ions by zinc ions in the HAp structure. Ultrasound measurements showed that HApLF powders are less stable compared to 5ZnHApLF powders. This behavior could be explained by the substitution of calcium ions by zinc ions in the HAp structure. This behavior confirms previous studies, which showed that the substitution of calcium ions in the HAp structure can lead to defects without affecting the hexagonal HAp structure [26,27,29,30].

These stability studies of lyophilized powders are important in view of the possibility of obtaining dense and reliable products [14,36,37,38]. Powder stability studies by ultrasound measurements are all the more important knowing that agglomerates are difficult to remove by the conventional dry powder pressing process [12,13,14,37,38]. Thus, ultrasound studies on the stability of lyophilized HAp and 5ZnHAp powders play an important role, providing us with information about the stability of the suspension.

**Figure 7:**
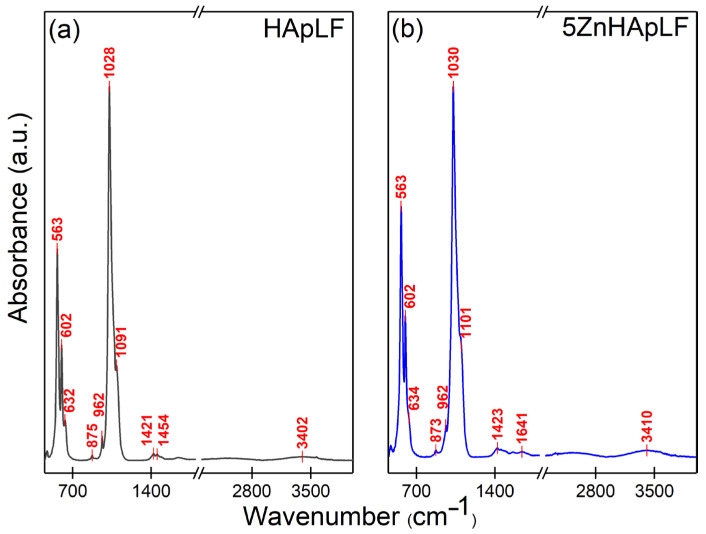
FTIR spectra of the HApLF (**a**) and 5ZnHApLF (**b**) samples.

Information regarding the functional groups present in the analyzed samples, as well as data about their purity and crystallinity, were obtained from Fourier Transform Infrared Spectroscopy studies. In Figure 7, the FTIR spectra of HApLF and 5ZnHApLF samples are revealed, recorded between 450 and 4000 cm^−1^. 

Thus, it could be observed that the FTIR spectra of HApLF are dominated by the intense band centered at 1028 cm^−1^, which corresponds to the asymmetric stretching vibration of the phosphate group (PO_4_^3−^) in the HAp structure [21]. Moreover, the other maxima observed at 1091 cm^−1^ are characteristic of the asymmetric stretching vibration of the phosphate group (PO_4_^3−^) [21]. The intense bands observed at 563 cm^−1^ and 602 cm^−1^ could be attributed to the bending vibration of the phosphate group (PO_4_^3−^) in the HAp structure [21]. The presence and intensity of these bands provide important information about the structure and crystallinity of HAp. On the other hand, according to the studies reported by Sahadat Hossain, M. et al. [39], the clear separation of these bands, especially in the 563/602 cm^−1^ doublet, is a strong indicator of a well-crystallized HAp structure. The symmetric stretching vibration band (ν_1_) of the (PO_4_^3−^) group can be observed in the HAp spectrum at 962 cm^−1^. Around 632 cm^−1^, the libration vibration band of the (OH^−^) group from HAp appears. In the study conducted by Iconaru, S.L. et al. [21], the weak vibration band observed at 875 cm^−1^ was attributed to (HPO_4_^2−^) [21]. At the same time, the vibration bands found in the 1400–1500 cm^−1^ spectral region can be mainly attributed to the stretching vibration of the carbonate group (CO₃^2−^), which suggests the B-type substitution of carbonate in the HAp structure [21,39,40,41]. Another weak vibration band could be noticed at around 1641 cm^−1^. The presence of this band could be attributed to adsorbed water molecules on the HAp surface [42]. Moreover, the broad band around 3402 cm^−1^ is characteristic of the presence of adsorbed water molecules [39,40]. No other additional vibration bands are observed in either FTIR spectrum, suggesting sample purity. Could be observed that the functional groups present 5ZnHApLF are similar with the one observed in the HApLF sample. The doping of HAp with a low concentration of zinc ions (x_Zn_ = 0.05) induce a slight shift in the vibrational bands position in the FTIR spectra of 5ZnHApLF samples. These results are in concordance with the one previously reported by Iconaru, S.L. et al. [21]. In addition, the intensity of the main vibrational bands indicates that both samples are well crystallized [43], a result that is in good agreement with the one obtained from XRD studies.

**Figure 8:**
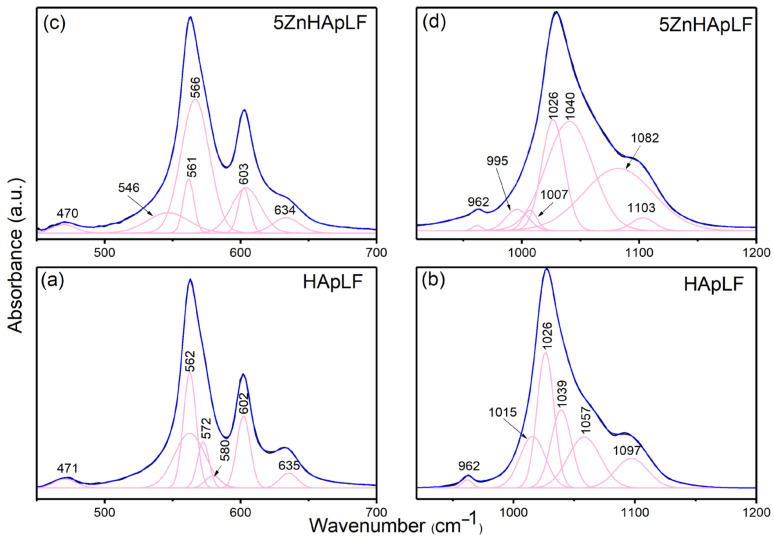
FTIR deconvoluted spectra of the ν_4_, ν₃, ν_2_, and ν₁ domains obtained for the HApLF (**a**,**b**) and 5ZnHApLF (**c**,**d**) samples.

The deconvolution of FTIR spectra of HAp provides detailed insight into the chemical composition, and modifications induced by substitutions, allowing for a more precise characterization of this biomaterial. Thus, in Figure 8, the deconvoluted FTIR spectra for two important spectral domains are presented: 450–700 cm^−1^ (the specific domain of the ν_2_ and ν_4_ [PO_4_^3−^] vibrations of the HAp structure) and 950–1200 cm^−1^ (the specific domain of the ν_1_ and ν_3_ vibrations of phosphate groups from the HAp structure). 

In Figure 8, the experimental and calculated contours are overlaid (blue line), revealing individual subbands (pink lines) identified through curve-fitting analysis. For the HApLF sample, seven components were needed in order to obtain a good fit in the 450–700 cm^−1^ spectral domain. Meanwhile, for the 950–1200 cm^−1^ spectral domain of Hap, the use of six subbands allowed for obtaining a satisfactory fit. For the 5ZnHApLF sample, seven components were needed for achieving a good fit in the 450–700 cm^−1^ spectral domain specific of ν_2_ and ν_4_ of the [PO_4_^3−^] groups. Nine subbands were needed in the 950–1200 cm^−1^ spectral region characteristic of ν_1_ and ν_3_ of the [PO_4_^3−^] groups. We observed that the main functional groups responsible for the absorbance of the HApLF and 5ZnHApLF samples in the 450–700 cm^−1^ and 950–1200 cm^−1^ spectral regions are represented by phosphate [PO_4_^3−^] groups from the HAp structure. More than that, in the 450–700 cm^−1^ spectral domain, along with the vibration bands characteristic of phosphate groups, the vibration band assigned to the hydroxyl group also appears (at around 634 cm^−1^). Moreover, a slight decrease in the individual component centered around 962 cm^−1^ in the case of 5ZnHApLF could be noticed in Figure 8b,d. This result it is in good agreement with the one reported by Iconaru, S.L. et al. [21].

**Figure 9:**
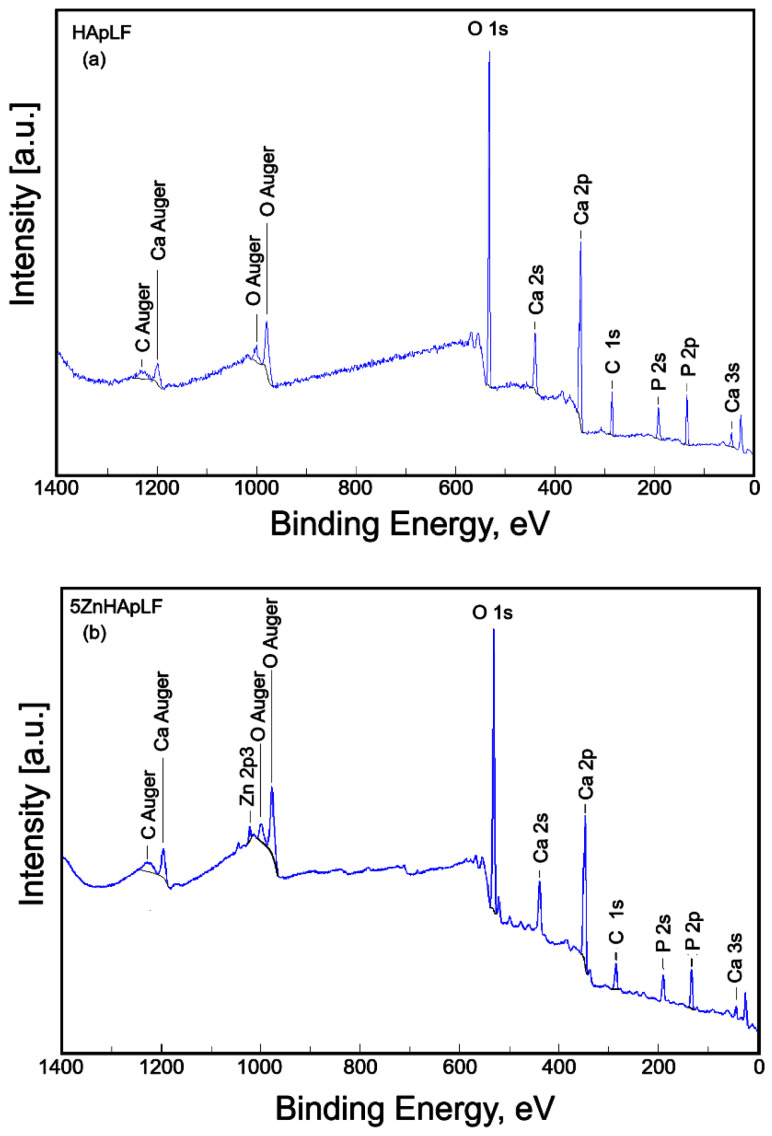
XPS survey spectra of the HApLF (**a**), 5ZnHApLF (**b**) samples.

The typical XPS survey spectra of the HApLF and 5ZnHApLF samples are presented in Figure 9. The XPS spectrum of the HApLF sample (Figure 9a) revealed the presence of constituent elements carbon (C), oxygen (O), calcium (Ca), and phosphorus (P). The XPS spectrum of the 5ZnHApLF sample (Figure 9b) also revealed the constituent element. In addition to the elements carbon (C), oxygen (O), calcium (Ca), and phosphorus (P), zinc (Zn) was also observed in the XPS spectrum of the sample 5ZnHApLF (Figure 9b). Table 2 presents the surface atomic composition (atomic %) of the HApLF and 5ZnHApLF samples.

**Figure 10:**
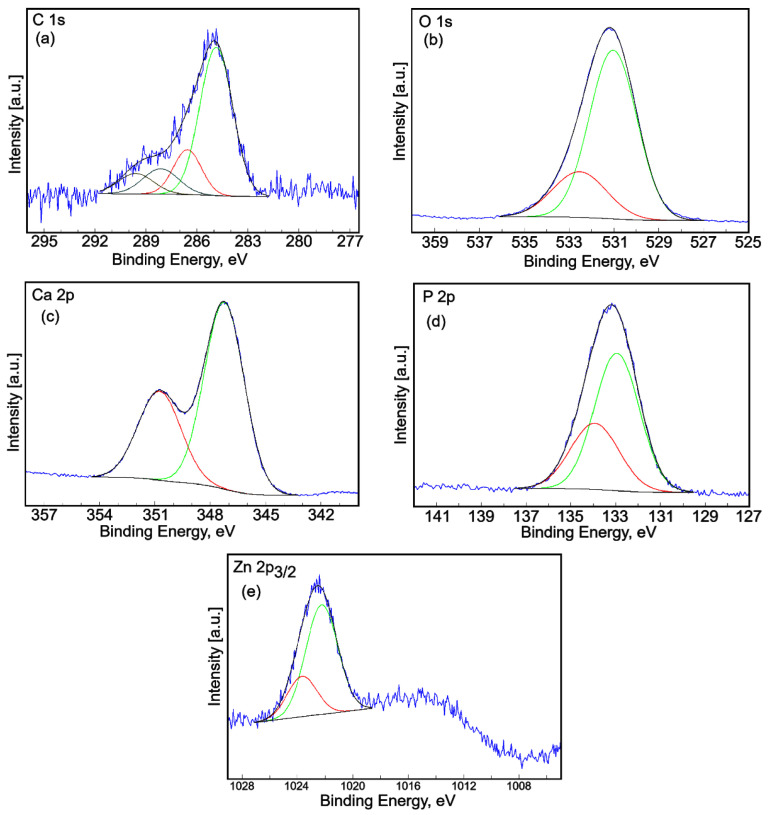
High-resolution XPS spectra of C 1s (**a**), O 1s (**b**), Ca 2p (**c**), P 2p (**d**), and Zn 2p3/2 (**e**) for the 5ZnHApLF sample.

The high-resolution XPS spectra of C1s, O 1s, Ca 2p, P 2p, and Zn 2p3/2 for 5ZnHApLF are shown in Figure 10. The high-resolution XPS spectrum of C1s for the 5ZnHApLF sample revealed four peaks (Figure 10a). The peak located at a binding energy (BE) of 284.86 eV was assigned to C–C single bonds [44]. The second peak, identified at a BE of 286.56 eV, was assigned to single bonds O–C–O [44]. The third peak, observed at a BE of 288.13 eV, was associated with the C=O double bonds [44]. The fourth peak, observed at a BE of 289.59 eV, was attributed to the –COOR-type contaminants [44,45].

The high-resolution XPS spectrum of O1s for the 5ZnHApLF sample is shown in Figure 10b. The high-resolution XPS spectrum of O1s shows two peaks. The first peak was observed at a BE of 531.04 eV, while the second peak was observed at 532.53 eV. The first peak was attributed to the oxygen in HAp and also includes C-O single bonds [46,47]. The second peak was assigned to the O=C–O and C=O bonds [46,47]. Figure 10c shows the high-resolution XPS spectra of Ca 2p for the 5ZnHApLF sample. The two peaks are located at Ca 2p 3/2 and Ca 2p 1/2 [48]. Two specific peaks were observed at a BE of 347.24 eV and 350.84 eV, respectively. The two specific lines (2p3/2 and 2p1/2) are spaced at approximately 3.6 eV. For the two peaks observed, the area ratio was close to 2:1. The high-resolution XPS spectra of P 2p for the 5ZnHApLF sample is presented in Figure 10d. The high-resolution XPS spectra of P 2p for the 5ZnHApLF sample exhibited two contributions [49]. The first contribution, identified with the specific 2p1/2 line, was located at a BE of 132.94 eV, while the second contribution, assigned to the specific 2p3/2 line, was presented at a BE of 133.84 eV. The two specific lines (2p3/2 and 2p1/2) are spaced at approximately 0.9 eV, with an area ratio of 2:1. The binding energy is specific to hydroxyapatite.

The high-resolution XPS spectra of Zn 2p3/2 for the 5ZnHApLF sample are presented in Figure 10e. The high-resolution XPS spectra of the P 2p region of the 5ZnHApLF sample exhibited two contributions. The first contribution was observed at a BE of 1022.26 eV, while the second contribution was identified at a BE of 1023.68 eV. The first contribution, located at a BE of 1022.26 eV, was assigned to the chemical energy bond of Zn-O [50]. The second contribution may be associated with the Zn^2+^ ions [51].

**Figure 11:**
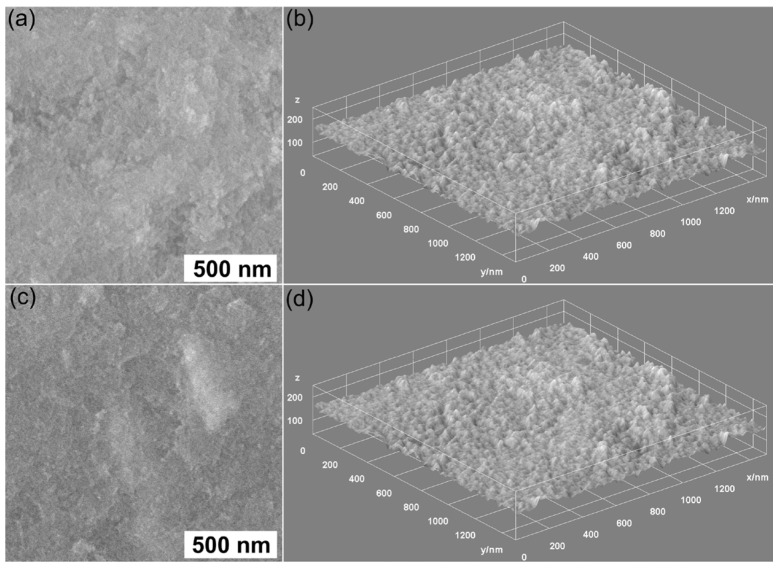
Two-dimensional SEM micrographs of the HApLF (**a**) and 5ZnHApLF (**c**) pellet surfaces and their corresponding 3D representations (**b**,**d**).

The morphology of HApLF and 5ZnHApLF was studied with the aid of SEM. Furthermore, complementary information about the sample morphology was also obtained by recording SEM micrographs of the pellet’s surfaces, obtained from the HApLF and 5ZnHApLF powders. The results of the SEM investigations are depicted in Figure 11.

The findings suggested that the surfaces of the pellets are homogenous and uniform, consisting of particles with nanometric sizes that exhibit a tendency to form conglomerates [52]. In addition, the 3D representation of the SEM images of the HApLF and 5ZnHApLF pellet surfaces also confirms the uniform and homogenous surface properties of the samples. The minor irregularities that are observed on the surface of the pellets could be attributed to the pellet fabrication process. Furthermore, the SEM images were used to quantify the surface roughness of the samples. The results obtained for the roughness parameter, R_RMS_, were 40.93 nm for HApLF and 79.48 nm for 5ZnHApLF.

**Figure 12:**
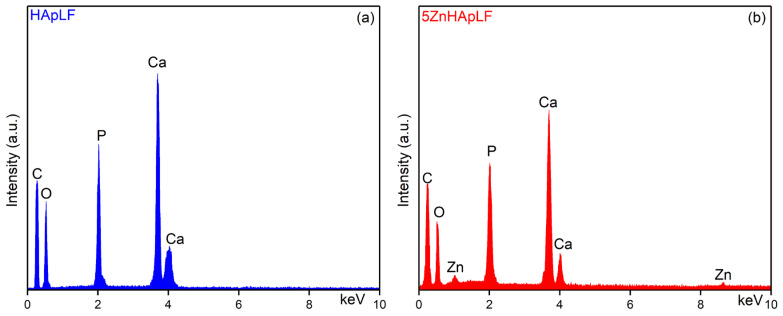
The EDS spectrum obtained for the HApLF (**a**) and 5ZnHApLF (**b**) samples.

Additional information regarding the chemical composition of HApLF and 5ZnHApLF was obtained by the EDS (Energy Dispersive Spectroscopy) method, and the results are presented in the Figure 12.

The main chemical elements that can be identified in the EDS spectrum of HApLF are calcium (Ca), phosphorus (P), and oxygen (O) (Figure 12a). In the case of the 5ZnHApLF sample, the EDS spectrum could be observed only the presence of Calcium (Ca), Phosphorus (P), Oxygen (O) and Zinc (Zn). The Carbon (C) line observed in both EDS spectra appears due to the carbon tape on which the samples were placed in order to be analyzed.

The atomic ratio Ca/P for stoichiometric HAp is 1.67. This ratio can vary slightly depending on the synthesis conditions (Table 3). In the case of HApLF, the value of the Ca/P ratio was 1.666. Furthermore, the value of (Ca + Zn)/P ratio was 1.679 for the 5ZnHApLF sample.

The results of the EDS studies do not reveal the presence of impurities or residual elements from the synthesis process in the analyzed samples, a fact that underlines the purity of the samples. The EDS results provide a semi-quantitative analysis of the chemical composition of the HApLF and 5ZnHApLF samples. Therefore, these results are in agreement and are also supported by the results of XPS studies.

**Figure 13:**
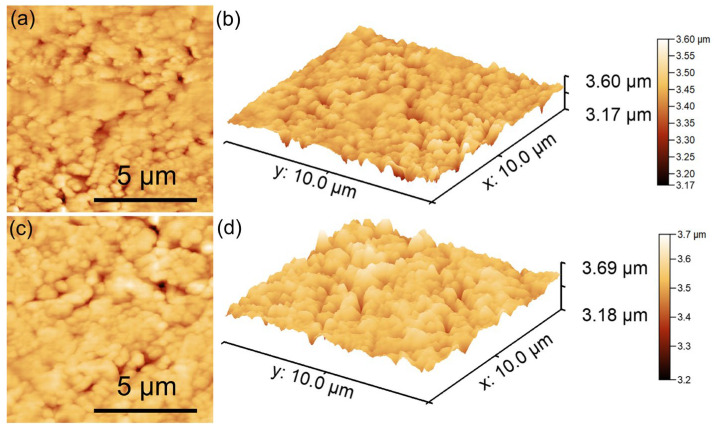
Two-dimensional AFM topographies of HApLF (**a**) and 5ZnHApLF (**c**) pellet surface on an area of 10 × 10 µm^2^ and their corresponding 3D representations (**b**,**d**).

The morphology of HApLF and 5ZnHApLF was further studied using the AFM technique. Details about the sample morphology were obtained by recording AFM topographies on surface areas of 10 × 10 µm^2^ of the pellets’ surface topography obtained from the HApLF and 5ZnHApLF samples. The results of the AFM studies are depicted in Figure 13.

**Figure 14:**
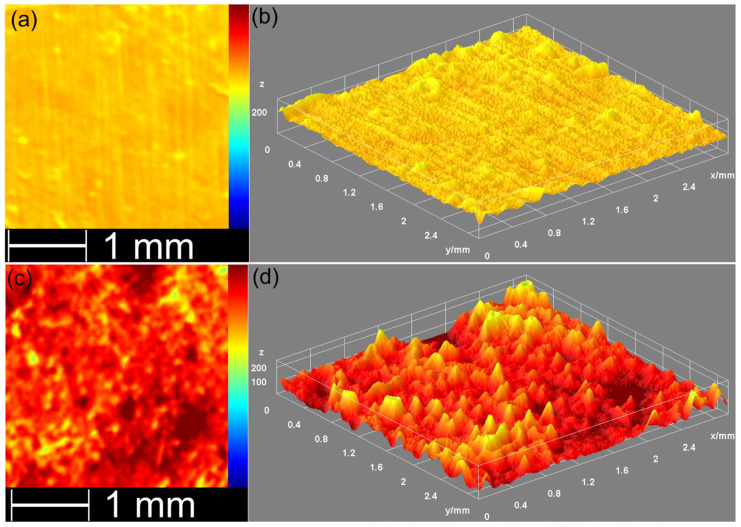
Evolution of the maximum of the signal reflected from the surface (C-scan, SAM) of HApLF (**a**) and 5ZnHApLF (**c**) pellet surface on an area of 3 × 3 mm^2^ and their corresponding 3D representations (**b**,**d**).

The AFM topographies of the HApLF and 5ZnHApLF reveals in both cases a surface having slight irregularities which could be attributed to the process used of obtaining the pellets. Furthermore, the AFM data shows that both in the case of HApLF and 5ZnHApLF pellet’s surfaces, a homogeneous distribution of agglomerated particles was observed. More than that, the 2D AFM topographies highlighted that the particles exhibit nanometric sizes. Both the 2D surface topography of the investigated surface areas and their 3D representation confirmed that the samples present a uniform and homogenous morphology consisting of particles having nanometric sizes and a tendency to form conglomerates. Moreover, slight irregularities on the pellet surface could be observed. Furthermore, the roughness parameter, R_RMS_, quantified for the area of 10 × 10 µm^2^ of the pellet surface topography in the case of HApLF was 45.37 nm, while in the case of 5ZnHApLF, it was 96.61 nm. The roughness parameter values indicate a uniform distribution of nanoparticle agglomerates across the pellet surface. These results are attributed to the presence of the zinc ions in the HAp matrix. The use of zinc ions as dopants for HAp significantly influences the material’s surface roughness. This is of great interest due to the fact that the roughness of a material is an essential factor in biomedical applications, especially for the ones related to bone tissue engineering. The incorporation of zinc ions into the HAp matrix disrupts the crystal structure and determine variations in grain size, thus promoting a rougher surface morphology [53,54,55,56]. Acoustic microscopy analysis (see Figure 14) confirmed this result.

The increase in roughness has an important role in enhancing cellular attachment, proliferation, and differentiation by providing a more favorable microenvironment for osteoblast adhesion. Additionally, Zn, as a vital trace element, offers biological benefits by promoting osteogenic activity. Therefore, Zn-doped HA not only improves the bioactivity of the material but also increases its surface roughness, which collectively contribute to enhanced osseointegration when applied as a bone substitute or coating on implant surfaces. These results are in good agreement with literature findings and supports the hypothesis that the presence of the dopant in the HAp matrix leads to an increase in the material’s roughness. The presence of a dopant causes lattice distortions, leading to an increase in surface defects, as these distortions extend to the material’s surface. In the study conducted by Ahmed et al. [55], it was reported that the physical and biological properties of the nanofibers were significantly influenced by variations in the dopant ion content within the minor ceramic component. They suggested that the precise addition of ionic dopants in such core/shell composites could create biomaterials with customizable properties, making them well-suited for diverse biomedical applications. The 3D topographic representation shows a generally smooth surface, with minor irregularities formed during the pellet fabrication process. These surface features, resulting from the pressing process, could enhance the adhesive properties of the material, supporting its potential for biomedical applications [54]. Surface roughness measurement is highly influenced by the technique used, as various methods operate on different measurement scales. Roughness values for the same surface can vary significantly when measured with different techniques and equipment settings. Generally, higher resolution in roughness measurements tends to yield higher roughness values, as resolution depends on the line or pixel density of the measurements and the probe size used [57,58].

Additional information regarding the surface properties of HApLF and 5ZnHApLF was achieved by quantifying the surface roughness parameters R_a_ and R_q_ (R_RMS_) of the HApLF and 5ZnHApLF pellet surfaces from SEM and AFM studies. The results of the studies are depicted in Table 4. The data suggest that the values of the roughness average (R_a_) and the root mean square (RMS) roughness (R_q_) obtained from the two complementary techniques were 23.61 (SEM) and 34.19 nm (AFM), in the case of (R_a_), and 40.93 (SEM) and 45.97 (AFM) nm for (R_q_), in the case of the HApLF sample. In addition, the values obtained for 5ZnHApLF from SEM and AFM studies were 51.49 and 80.03 nm for R_a_ and 79.48 and 96.61 nm for R_q_. The differences obtained from the study regarding the determination of the roughness parameters by various techniques could be attributed to the limitations of the instruments in acquiring the images, as well asto their quality. Nonetheless, the values for the roughness parameters obtained from the two complementary techniques did no exceed 96.61 in the case of R_q_ and were lower than 23.61 for R_a_, suggesting a homogenous and uniform surface with minimum roughness. The data also show that the R_q_ parameter exhibited higher values compared to the R_a_ values for both samples and in the case of both techniques. These findings indicate that the investigated surfaces have more pronounced peaks and valleys, which can enhance their functionality for specific applications.

The surface roughness parameters R_a_ and R_q_ are usually used to quantify the texture of a surface. The parameter R_a_, or arithmetical mean height, represents the average absolute deviation of surface heights from the mean plane, offering a straightforward indicator of surface roughness. The parameter R_q_, or root mean square height, depicts a more statistical approach by determining the square root of the mean of the squared deviations from the mean plane. While both parameters are used to describe surface roughness, R_q_ tends to be more sensitive to larger peaks and valleys because squaring emphasizes these extreme features. In practice, if R_q_ is significantly higher than R_a_ for the same sample, it indicates the presence of pronounced peaks or deep valleys, suggesting a more irregular surface, with occasional high deviations from the mean. This distinction can be important in applications where the surface consistency or peak management is important, as surfaces with higher R_q_ values may impact functionality, wear, or adhesion differently compared to those with closer R_a_ and R_q_ values. When a surface has an R_q_ (root mean square roughness) value higher than its R_a_ (average roughness) value, it can offer unique benefits in particular applications. A higher R_q_ parameter value indicates that the investigated surface has more distinct peaks and valleys, which can help enhance its functionality for particular applications. In applications that require an improved adhesion, a surface with greater topographical variation has the ability to provide more “anchor” points, thus enabling the coatings or adhesives to bond more intensely. Similarly, in the case of fluid retention, such as lubrication or cooling, the deeper valleys can act as micro-reservoirs, helping retain the liquids and improving their lubrication efficiency. This type of surface can also be advantageous in reducing light reflection or scattering, which is beneficial in optical applications where controlled roughness improves anti-glare properties. Thus, a surface with a higher R_q_ than R_a_ can be an asset when precise material interactions are desired, making it valuable across fields that rely on optimized surface engineering [57,58,59,60,61,62,63,64].

AFM and SEM together provide a comprehensive view of surface roughness by pairing nanoscale precision with broader topographical analysis. AFM is a technique that is particularly effective for obtaining quantitative roughness data, such as root mean square (R_RMS_) roughness, at the nanometer scale. This allows the uncovering of subtle irregularities and fine textures, which are essential for applications requiring detailed surface characterization. On the other hand, SEM could offer a much larger field of view, allowing the examination of microscale features and structural patterns across the pellet surfaces, such as cracks, voids, and particle clusters, which could influence bulk properties such as porosity and mechanical strength. By combining AFM’s high-resolution roughness measurements with SEM’s expansive morphological perspective, a comprehensive understanding of surface roughness, effectively connecting fine surface details with broader structural features could be achieved. This combined approach is particularly valuable in fields such as catalysis, drug delivery, and coatings, where both nanoscale and microscale characteristics critically influence the material’s performance. The results of SEM and AFM studies both emphasize the fact that the presence of zinc ions in the 5ZnHApLF leads to a higher roughness of the samples compared to HApLF. The increased roughness of 5ZnHApLF compared to that of HApLF highlights the critical role of surface texture in enhancing the material’s suitability for biomedical applications. The higher roughness value of 5ZnHApLF provides additional surface area and micro-topographical features, which are advantageous for cell adhesion, proliferation, and differentiation. This textured surface more closely resembles the natural extracellular matrix, supporting improved osteoblast attachment and bone integration, which are essential in bone tissue engineering. Additionally, the enhanced roughness can improve implant stability by increasing friction and mechanical interlocking with the surrounding tissue. Thus, the elevated roughness of 5ZnHApLF makes it a promising material with tailored properties for advanced biomedical applications, particularly in bone-related implants.

The hardness of the HApLF and 5ZnHApLF pellets was evaluated with the Vickers method, and the results are revealed in Table 5. Thus, the value obtained for the HApLF pellet hardness was 2.8 GPa. In the meantime, it could be noticed that the value of hardness obtained for the 5ZnHApLF pellet is higher as compared to that of HApLF. For the 5ZnHApLF pellets, a hardness value equal to 3.1 GPa was obtained. Thus, our results are in good agreement with previous studies, which showed that the addition/doping of HAp with various ions induces an enhancing of its mechanical features [65,66,67,68,69,70,71,72,73,74,75,76].

Synthetic HAp has shown promising results in bone tissue engineering, but it suffers from significant drawbacks, such as poor mechanical strength, low fracture toughness, and brittleness, making it unsuitable for load-bearing applications [65,66,67,68,69,70,71,72]. To overcome these limitations, researchers have explored various methods to enhance its physico-chemical and biological properties. The most effective approach involves modifying the HAp structure through doping with various ions [65,66,67,68,69,70,71,72]. According to previous studies [65,66,67,68,69,70,71,72,73], this process of incorporating various ions into HAp structure improves its mechanical properties and biological activity. These modifications aim to address the inherent weaknesses of synthetic HAp and expand its potential applications in bone tissue engineering [67,68,69,70,71,72,73,74,75]. Uysal, I. et al. [74] also reported that the doping of HAp with Zn ions enhanced the mechanical properties compared to those found for pure synthetic HAp [74]. Furthermore, the research conducted by Bensalem, A., and coworkers [75] demonstrates that incorporating silver and zinc oxide into HAp significantly enhances its bactericidal and mechanical features [75]. Moreover, the results reported by Lu, H. and colleagues [76] underline that the freeze-drying method significantly enhances the mechanical properties of HAp ceramics compared to ones of Hap that is conventionally heat-dried [76].

**Figure 15:**
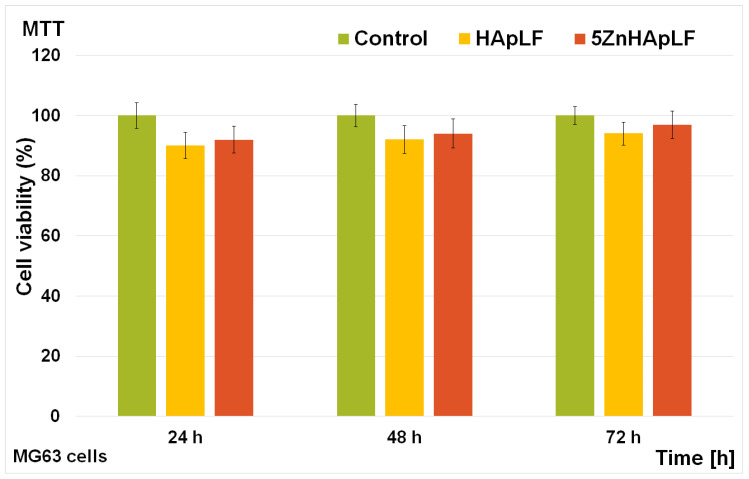
Cell viability of MG63 cells incubated with HApLF and 5ZnHApLF for 24, 48, and 72 h. The results are represented as mean ± standard deviation (SD) and are expressed as percentages relative to the control (100% viability).

The cytotoxicity of the HApLF and 5ZnHApLF pellets was assessed with the help of the MTT assay. The MG63 cell viability was determined for three different periods of incubation (24, 48 and 72 h). The results of the MTT assays are depicted in Figure 15. The data are graphically illustrated as the mean ± standard deviation (SD) from three experiments having the cell viability represented as percentage of the control, which was considered 100%.

**Figure 16:**
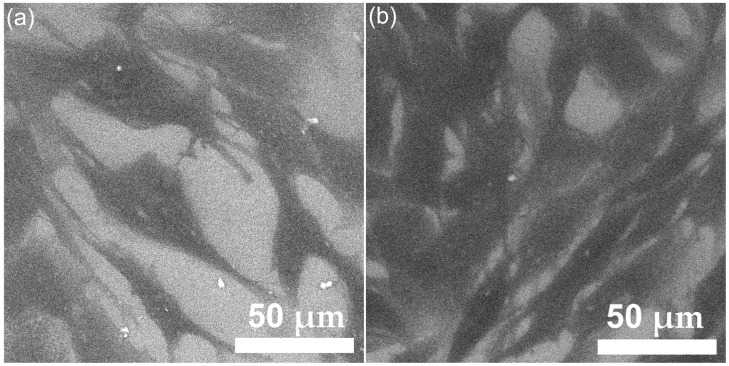
SEM micrographs of MG63 cells attachment on the surfaces of HApLF (**a**) and 5ZnHApLF (**b**) pellets after 72 h of incubation.

The findings highlighted that the viability of the MG63 cells was comparable to the one of the control for all the tested intervals, thus revealing good biocompatibility. Furthermore, the results of the MTT assay presented in Figure 15 revealed that the viability of HApLF remained above 90% for 24 h even in the early stages of incubation and increased gradually with the increase in the incubation time to 92% after 48 h, reaching 94% after 72 h. On the other hand, the cell viability of MG63 cells incubated with 5ZnHApLF after 24 h was 92% and also increased with the incubation time, reaching 94% and 97% after 48 and 72 h, respectively. These results show that both the HApLF and 5ZnHApLF pellets exhibited a good biocompatibility towards MG63 cells. These results align with previous reported studies on HAp and ZnHAp biocomposites, which consistently highlighted the favorable biological properties of these types of materials [15,77,78,79,80,81,82,83,84,85,86]. The presence of zinc ions in the matrix of HAp has the ability to enhance both cell proliferation and differentiation, thus supporting the viability of the cultured cells. The inclusion of zinc, which is well known as being an essential trace element, confers HAp antimicrobial properties, helps promote osteoblast proliferation, and also supports the enzymatic activity, which is crucial for bone remodeling and mineralization [77,81,85]. The results obtained regarding the toxicity of the HApLF and 5ZnHAppLF pellets are in good agreement and are corroborated by the studies reported by Tank et al. [77], which demonstrated ZnHAp’s biocompatibility with MG63 osteoblast cells. Similar results were also achieved and reported by Thian et al. [81], who highlighted its excellent bioactivity towards mesenchymal stem cells (MSCs) derived from human adipose tissue. Additionally, Radovanović et al. [83], in their studies on the biological properties of zinc-doped hydroxyapatite (ZnHAp) in MRC-5 fibroblast cells reported its excellent biocompatibility. Moreover, Thian et al. [81] demonstrated in their study that by incorporating Zn^2+^ ions into the hydroxyapatite matrix, the bioactivity of HAp could be considerably enhanced. These findings align with the present study, emphasizing that ZnHAp exhibits excellent biocompatibility and supports cell viability and proliferation across various cell types, including human osteoblasts, mesenchymal stem cells, and fibroblasts. These results confirm HApLF and 5ZnHApLF as promising candidates for the development of future applications in bone grafts, dental implants, and drug delivery systems targeting bone-related conditions. Complementary information regarding the adhesion and development of MG63 cells on the surface of HApLF and 5ZnHAppLF pellets was acquired through SEM visualization. For this purpose, after 72 h of incubation, the adhered MG63 cells were fixed on the pellet surfaces with glutaraldehyde dehydrated with ethanol and air-dried before SEM observation. The results of the SEM studies are depicted in Figure 16.

Information about the interaction of MG63 osteosarcoma cells with the HApLF and 5ZnHApLF pellets is crucial for the evaluation of the material’s potential for being used for biomedical applications. Therefore, SEM has been acknowledged as an invaluable tool in the studies regarding cell-surface interactions at a microstructural level. SEM studies provide valuable information about cell adhesion, spreading, and morphology. The SEM micrographs depicted in Figure 16a showed that when cultured on HApLF pellets, MG63 cells exhibited good adherence. More than that, the micrographs revealed that the cells firmly attached to the surface of the HApLF pellet. Furthermore, the SEM images depicted in Figure 16b also highlighted that the 5ZnHApLF exhibited an excellent adherence. The SEM visualization suggested that, when cultured on 5ZnHApLF pellets, MG63 cells exhibited a significantly enhanced adherence compared to HApLF. The micrographs highlighted that there was an improvement in cell attachment to the 5ZnHApLF surface, evidenced by the presence of extensive filopodia and lamellipodia spread over the pellet’s surface. The presence and wide spread of these structures, which are crucial for the anchoring and intracellular signaling of the cells, indicate a superior physical interaction between the MG63 cells and the pellets’ surface, suggesting that the zinc-doped surface provides an optimal environment for MG63 cellular attachment. Furthermore, the SEM images revealed that the adhered cells on the surface of both HApLF and 5ZnHApLF pellets exhibited a pronounced flattened morphology having widespread cytoplasmic extensions. These characteristics highlight a robust and healthy adhesion on the studied surfaces. More than that, the SEM images highlighted an enhanced spreading across the 5ZnHApLF surface, which emphasize the material’s advanced biocompatibility and improved osteoconductive potential compared to the HApLF pellet’s surface. These results could be attributed to the increased roughness of the 5ZnHApLF compared to HApLF. The increase in roughness, influenced by zinc incorporation, most likely contributed to the improvement in cell attachment by providing additional anchoring sites and favorable microenvironments [87,88,89,90]. These properties are significant for the cellular processes, such as proliferation, osteogenic differentiation, and extracellular matrix deposition, all being essential for biomedical applications. Thus, the SEM findings evidence the superior MG63 cell adherence to 5ZnHApLF and emphasizes the material’s improved efficacy compared to HApLF. In addition, both the SEM results, as well as the MTT assay, support the potential of these materials in biomedical applications, including implants and bone graft substitutes. These findings pave the way for further optimization of zinc-doped apatite surfaces to maximize biological performance.

**Figure 17:**
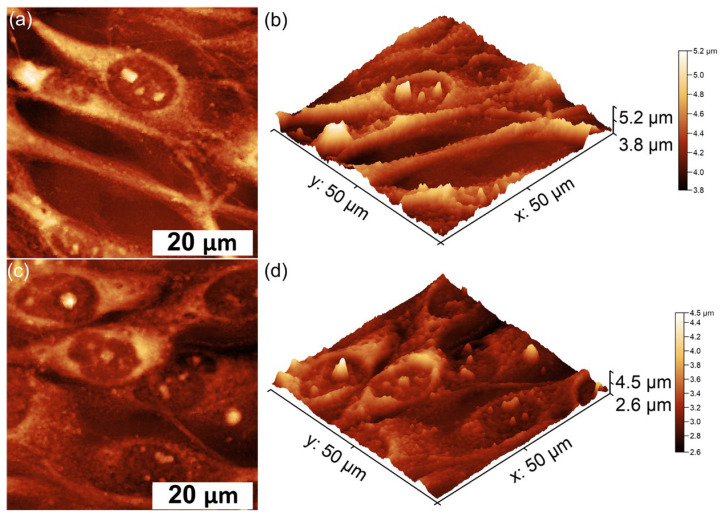
Two-dimensional AFM topography of MG63 cells after 72 h of incubation with HApLF (**a**) and 5ZnHApLF (**c**) pellets, along with their 3D representation (**b**,**d**).

The development and adherence of the MG63 cells, incubated for 72 h on the surface of HApLF and 5ZnHApLF pellets, were also examined by AFM. The AFM topographies of MG63 cell adhesion on the surfaces of the HApLF and 5ZnHApLF pellets were acquired over a 50 × 50 µm^2^ area under normal atmospheric conditions and at room temperature. The 2D topographies and their corresponding 3D representations after 72 h of incubation revealed the presence of distinct morphological features in MG63 cells adhering to the surfaces of HApLF and 5ZnHApLF pellets. These topographical analyses provide valuable insights into cell–material interactions and the influence of different surfaces on cellular behavior. The AFM results depicted in Figure 17 demonstrate that MG63 cells adhered successfully to both tested surfaces.

For the HApLF sample, the AFM topographies highlighted that the adhered cells retained their typical elongated, fibroblastic features without presenting any significant morphological changes [91,92,93,94,95,96]. The 3D representations further supported these observations, highlighting the alignment and distribution of cells on the surfaces. Moreover, both the 2D AFM topographies and their 3D representation indicated that MG63 cells spread uniformly across the surface of the 5ZnHApLF pellet, forming a monolayer of cells with an elongated and aligned fibroblastic morphology. This spreading behavior is characteristic to favorable cell–material interactions and highlight that the surface of the 5ZnHApLF pellet provides an optimal environment for cell attachment and growth. These findings emphasize the importance of surface properties in regulating cell morphology and could provide important insights for the future development of biomaterials for applications in tissue engineering and regenerative medicine. The AFM topographies showed that while the surface of the HApLF pellet exhibited a smooth and uniform topography, the surface of the 5ZnHApLF pellet exhibited a greater roughness due to the incorporation of zinc ions. These increase in the surface’s roughness is beneficial to cell attachment due to the fact that has the ability to provide additional anchoring points, enhancing the adsorption of proteins critical for cell attachment. Surface characteristics always play an important role in influencing the biological response of cells to biomaterials. The results of the AFM studies regarding the MG63 cells adherence and proliferation on the surface of HApLF pellets showed that the MG63 cells adhered moderately and exhibited a well-spread morphology but presented only limited filopodia extensions. On the other hand, the AFM findings revealed that the surface of 5ZnHApLF pellets facilitated a more pronounced cell adhesion, with MG63 cells showing extensive spreading and numerous filopodia interacting with the surface. The better adhesion and spread of MG63 cells on the surface of 5ZnHApLF pellets could be attributed to the synergistic effects of increased roughness and the bioactive properties of zinc, which is known to enhance osteogenic activity and cellular interactions. These results confirm the data obtained from the MTT biological assay, as well as the findings of the SEM observation, and are in good agreement with the literature [15,78,79,80,81,82,83,84,85,86,87]. These results emphasize the potential of 5ZnHApLF for use in future biomedical applications. The results regarding the enhanced MG63 cell adhesion on 5ZnHApLF, compared to HApLF, suggest that the presence of zinc ions improves not only the physical properties of HAp but also its biological compatibility. By promoting better cell–material interactions, 5ZnHApLF could lead to improved osteointegration and bone regeneration, making it a promising candidate for advanced orthopedic and dental implants.

**Figure 18:**
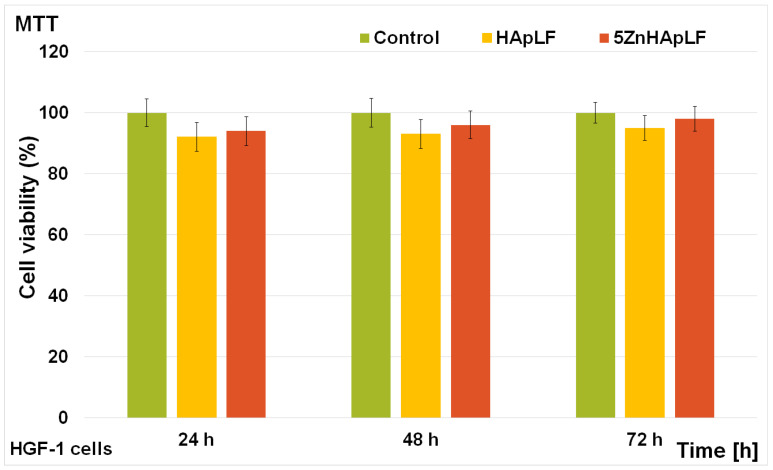
Cell viability of HGF-1 cells incubated with HApLF and 5ZnHApLF for 24, 48, and 72 h. The results are represented as mean ± standard deviation (SD) and are expressed as percentages relative to the control (100% viability).

Additional information regarding the cytotoxicity of HApLF and 5ZnHApLF pellets was obtained using the MTT assay, focusing on the viability of the human gingival fibroblasts (HGF-1) at different incubation periods, of 24, 48, and 72 h. The results of the MTT assay are depicted in Figure 18, and are presented as the mean ± standard deviation (SD) of three independent experiments, with cell viability expressed as a percentage relative to the untreated control, set as 100%. The findings of the cell viability assay revealed that both HApLF and 5ZnHApLF exhibited high biocompatibility, with cell viability consistently exceeding 92% across all tested time intervals. In the case of HApLF pellets, the HGF-1 cell viability was 92% after 24 h and increased to 93% and 95% at 48 and 72 h, respectively. Similarly, 5ZnHApLF displayed a viability of 94% at 24 h, further rising to 96% and 98% at 48 and 72 h. These results are in good agreement with the ones obtained for the MG63 cells and with previous reported studies on the cytotoxicity of HAp and zinc-doped HAp samples [15,77,78,79,80,81,82,83,84,85,86].

The findings of this evaluation suggest that the inclusion of zinc ions has the ability to enhance cell viability, which is consistent with previous reported studies that highlight zinc’s role in promoting cell proliferation and differentiation. The incorporation of zinc into HAp matrices is known to confer antimicrobial properties, enhance osteoblast activity, and support enzymatic processes essential for tissue remodeling and mineralization [15,77,78,79,80,81,82,83,84,85,86]. Zinc is an essential trace element that has a crucial role in various physiological processes, such as cellular signaling and protein synthesis. Its incorporation into HAp (ZnHAp) has the ability to improve the biocompatibility and osteoconductive potential of HAp by enhancing protein adsorption and providing a favorable microenvironment for cell attachment. Previous studies have shown that ZnHAp materials could enhance the proliferation and differentiation of various cell types, including fibroblasts, osteoblasts, and mesenchymal stem cells (MSCs) [77,81,83]. These finding aligns well with the results obtained from the cell viability assays against MG63 and HGF-1 cells, which indicated cell viability on 5ZnHApLF pellets compared to HApLF pellets, highlighting zinc’s significant role in improving Hap’s biological performance.

**Figure 19:**
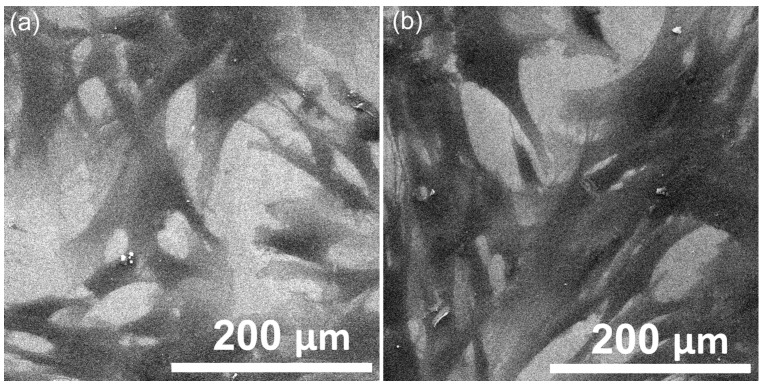
SEM micrographs of HGF-1 cells attachment on the surface of HApLF (**a**) and 5ZnHApLF (**b**) pellets after 72 h of incubation.

Complementary studies on the HGF-1 cell adherence and development were conducted using SEM and AFM analyses. The SEM and AFM studies were conducted to examine the interaction of HGF-1 cells with the surfaces of the HApLF and 5ZnHApLF pellets. After 72 h of incubation, SEM imaging (Figure 19) demonstrated that both materials exhibited excellent cell adhesion, having an enhanced attachment, observed in the case of 5ZnHApLF surfaces. The HGF-1 cells cultured on HApLF surfaces also exhibited good adherence, and spreading across the surface with moderate filopodia extension was also observed. Conversely, the cells adhering to the 5ZnHApLF surfaces showed an extensive filopodia and lamellipodia presence, which is indicative of an improved physical interaction and robust cell-surface adhesion.

**Figure 20:**
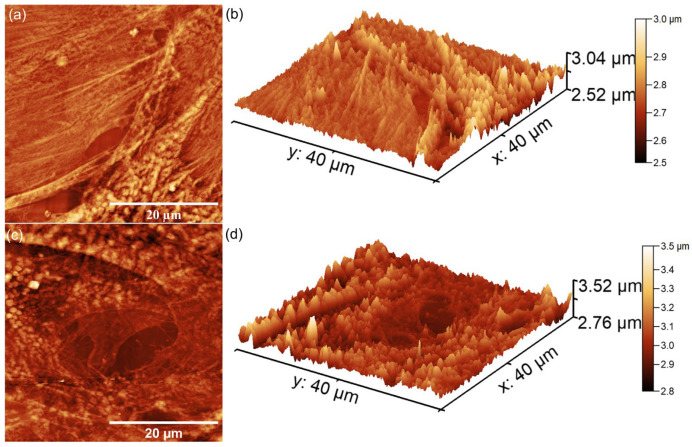
Two-dimensional AFM topography of HGF-1 cells after 72 h of incubation with HApLF (**a**) and 5ZnHApLF (**c**) pellets, along with their 3D representation (**b**,**d**).

Furthermore, the AFM topographies depicted in Figure 20 revealed the distinct morphological features of the HGF-1 cells adhering to the pellet’s surface. While HApLF exhibited a smooth surface, 5ZnHApLF displayed increased roughness due to zinc incorporation. This enhanced the surface texture, providing additional anchoring sites, thus promoting protein adsorption and facilitating cell attachment and spreading. These findings are in good agreement with the SEM results, emphasizing the superior biocompatibility and osteoconductive potential of 5ZnHApLF. The AFM 2D topographies indicated that the cells adhered to the surfaces of HApLF and 5ZnHApLF displayed patterns characteristic to the cellular morphology of fibroblast cells, showing flattened morphologies that ranged from spindle-like to tile-like forms [97,98,99]. Furthermore, both the 2D AFM topographies and their 3D representations demonstrated that after a 72 h incubation period, the fibroblast cells exhibited excellent adherence to the surfaces of the HApLF and 5ZnHApLF pellets. The results obtained by AFM investigation are in good alignment with the results of the MTT cell viability assays and SEM observations and suggest that both HApLF and 5ZnHApLF pellets do not exhibit any cytotoxic effects against the tested cells, making them considerable candidates for being used in the development of biomedical devices.

Additionally, the 2D AFM topographies highlighted that the cells adhered and spread effectively across the surfaces of the studied surfaces. The observed increase in both MG63 and HGF-1 cell adhesion and viability on 5ZnHApLF surfaces highlights the importance of zinc ions in enhancing HAp biological properties. The superior cell adhesion and proliferation observed on 5ZnHApLF pellets suggest that zinc’s presence enhances HAp’s ability to facilitate osteointegration and soft tissue healing. This aligns well with the reports from the literature, highlighting the synergistic effects of surface topography and zinc doping in promoting cellular responses essential for successful biomedical applications [15,78,79,80,81,82,83,84,85,86,87].

The incorporation of zinc ions into the HAp matrix has been shown to enhance both its biological and mechanical properties, making the resulting composites promising materials for bone tissue engineering applications. The reported studies indicated that low concentrations of zinc in the HAp matrix are non-cytotoxic and highly beneficial for bone cells, having the ability to promote cellular proliferation and differentiation, which are crucial for bone regeneration therapies [80,100]. Additionally, it has been reported that zinc-doped HAp exhibits enhanced bioactivity and antibacterial properties compared to pure HAp, thus being able to reduce the risk of infection in implanted materials and improve the overall biocompatibility of the implants [80,100].

The increase in R_a_ values, representing surface roughness, as measured by atomic force microscopy (AFM), depicted in Table 4, exhibited a significantly influence on the adherence of MG63 osteoblast-like cells and HGF-1 gingival fibroblasts. The enhanced surface roughness provided topographical scaffolds that permit multiple layers of cells to proliferate, which promoted cell anchorage and spreading. In this context, the zinc ions also played a significant role by synergistically enhancing the cellular behavior through their bioactive properties, including promoting osteogenic activity and supporting cell attachment. For MG63 cells, higher R_a_ values combined with zinc ion release facilitate adhesion, proliferation, and differentiation, fostering bone tissue integration. A similar behavior was observed in the case of HGF-1 cells, which benefited from the moderate roughness as well as the presence of zinc ions, which enhanced the adhesion and development of the cells onto the surface of 5ZnHApLF pellets. Nevertheless, an excessive roughness could lead to a disruption of uniform cell spreading and also interrupt cellular interactions, emphasizing the importance of controlled surface modifications and the strategic incorporation of zinc ions to optimize cellular responses. By modifying the surface roughness and incorporating bioactive elements, zinc-doped HAp materials not only improve cell–material interactions but also support cellular functions that are critical for tissue repair and regeneration. These characteristics make 5ZnHApLF a promising candidate for applications in dental implants, periodontal therapies, and bone graft substitutes.

**Figure 21:**
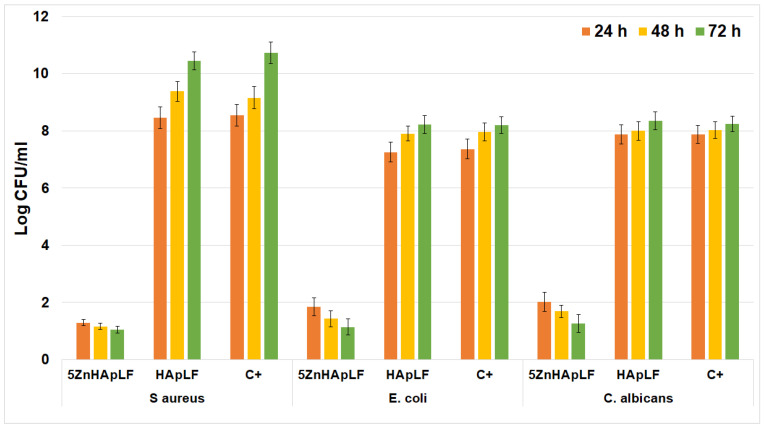
The graphical representation of the Log colony-forming units (CFU)/mL of *S. aureus* ATCC 25923, *E. coli* ATCC 25922, and *C. albicans* ATCC 10231 at different incubation times with HApLF and 5ZnHApLF. The results are expressed as means ± standard error of the mean from four independent experiments.

The antimicrobial activity of HApLF and 5ZnHApLF against *Escherichia coli*, *Staphylococcus aureus*, and *Candida albicans* was evaluated using the colony-forming unit (CFU) assay for three different incubation times (24, 48, and 72). The results are presented in terms of CFU reduction and provide information on the effectiveness of HAp and 5Zn-HAp as antimicrobial agents. The studies were conducted for 24 h, 48 h, and 72 h, and the antimicrobial properties of HApLF and 5ZnHApLF were quantified by assessing the inhibitory effects on the colony-forming unit (CFU) values of the microbial strains, compared to a free microbial strain used as the control (C+). The results from three independent experiments are presented in Figure 21 as mean ± SD.

The results of the antimicrobial assays demonstrated a significant reduction in the bacterial growth for *E. coli* exposed to 5ZnHApLF compared to pure HApLF. While HApLF did not exhibit any antimicrobial activity, the incorporation of zinc ions significantly enhanced its antimicrobial performance. Zinc is well known for its bacteriostatic and bactericidal properties and has the ability to disrupt bacterial membrane integrity and also to interfere with enzymatic processes, resulting in a reduced viability of *E. coli*. The CFU count for 5ZnHApLF demonstrated a considerable reduction compared to the control group, confirming its superior antibacterial efficacy. In the case of *S. aureus*, which is a Gram-positive bacterium having a thicker peptidoglycan layer, 5ZnHApLF also exhibited an enhanced antibacterial activity compared to HApLF. The results indicated a significant reduction in CFU for 5ZnHApLF, whereas HAp demonstrated no effects on the bacterial growth. The antimicrobial activity of 5ZnHApLF can be attributed to the zinc ion’s ability to disrupt the bacterial cell wall synthesis and to induce oxidative stress, which are particularly effective against Gram-positive bacteria. The observed reduction in CFU for 5ZnHApLF was significant compared to the control, emphasizing its broad-spectrum activity. In the case of *C. albicans*, a fungal pathogen, 5ZnHApLF, showed a considerable antifungal activity compared to HApLF, which exhibited little to no effect on the fungal growth. In this case, the antifungal mechanism of zinc is hypothesized to involve the disruption of fungal cell membrane integrity and interference with essential metal ion homeostasis within the fungal cells. The CFU count for *C. albicans* exposed to 5ZnHApLF decreased considerably, highlighting the potential of 5ZnHApLF to be considered as an effective antifungal agent. The results of this study are in good agreement with existing literature on the antimicrobial properties of zinc-doped materials [5,20,70,101,102]. Zinc ions are known to exert antimicrobial effects through multiple pathways, including membrane disruption, generation of reactive oxygen species (ROS), and interference with microbial metabolic pathways. The incorporation of zinc into the HAp matrix ensures a sustained release of antimicrobial agents, providing prolonged protection against microbial colonization [5,20,70,101,102]. The findings of the antimicrobial assays revealed that while HApLF showed no antimicrobial activity across all tested pathogens, 5ZnHApLF demonstrated significant efficacy against *E. coli*, *S. aureus*, and *C. albicans*. This behavior can be attributed to the synergistic effects of zinc ions, which enhance the antimicrobial properties of HAp. The enhanced activity of 5ZnHApLF against both Gram-negative and Gram-positive bacteria, as well as fungal cells, highlights it as a promising material for biomedical applications, particularly in infection-resistant implants and coatings. Furthermore, the effectiveness of 5ZnHApLF against both bacterial and fungal microbial cells highlights its versatility as a broad-spectrum antimicrobial material. This activity is highly relevant in clinical settings where infections are caused by multiple microorganisms. However, further studies are needed to optimize the zinc doping concentration, evaluate long-term biocompatibility, and also assess the material’s performance in in vivo models. Recent studies regarding the textural, structural, and biological evaluation of HAp doped with low concentrations of zinc have provided valuable information on the effects on cell viability in both prokaryotic and eukaryotic cells [15]. In their study, Predoi et al. [15] demonstrated that Zn:HAp exhibited a significant cytotoxicity against *S. aureus* while showing minimal cytotoxicity against *E. coli*. However, their findings also revealed that the tested Zn:HAp showed intense cytotoxic effects on hepatic cells. Similarly results reported by Popa et al. [103] in their structural and biological assessments of zinc-doped HAp nanoparticles concluded that Zn doping at the tested concentrations did not cause specific toxicity in either prokaryotic or eukaryotic cells. Their findings revealed that the activity of Zn:HAp nanoparticles against HepG2 cells was strongly influenced by particle size. On the other hand, Khan et al. [104] explored flower-shaped ZnO nanoparticles synthesized using a novel near-room-temperature approach, highlighting how the shape of nanoparticles affects their antibacterial and antifungal properties. In their evaluation of the antimicrobial activity of ZnO against *S. aureus*, *E. coli*, and *C. albicans*, Phaechamud et al. [105] reported that at low concentrations, ZnO inhibited the growth of *S. aureus* more strongly compared to *C. albicans* or *E. coli*.

Nanoscale drugs are intricate structures engineered at the nanoscale, offering significant potential in treating and targeting various diseases due to their numerous therapeutic benefits. Previous studies [106,107] highlight several advantages of nanomedicines, including the protection of biomolecules from degradation, enhanced solubility and bioavailability, reduced toxicity, and improved therapeutic efficacy. Due to these unique properties, several nanoscale products have recently received approval from the US Food and Drug Administration (FDA) and the European Medicines Agency (EMA) for medical use [108]. For a product to achieve commercial success, it must be both safe and highly effective. However, according to Kaur IP et al. [109], most nanoscale products fall short of these criteria. The limited clinical success of nanomedicines is primarily attributed to challenges in the synthesis process, reproducible manufacturing, screening, instability in biological environments, and batch-to-batch consistency [106,107,108,109,110]. Additionally, the specific physicochemical and biological properties that confer various benefits to nanoscale drugs also pose safety challenges. As indicated in previous studies [106,107,108,109,110], numerous obstacles must be overcome to achieve the successful scale-up of these novel nanoscale drugs, presenting exceptional challenges. The production of ZnHAp powders and pellets involves multiple steps, including homogenization, sonication, centrifugation, evaporation, pressing, drying, size reduction, lyophilization, and sterilization.

This research used lyophilization as a drying method for the first time in the process of obtaining HAp and 5ZnHAp powders with improved physicochemical and biological properties. Applying lyophilization after obtaining HAp and 5ZnHAp powders through an adapted coprecipitation method offers significant benefits by being able to preserve the material’s structural and chemical integrity. The lyophilization process enables the elimination of capillary stresses commonly encountered in conventional drying methods. On the other hand, the lyophilization process minimizes contamination, enhances sinterability, and enables the production of advanced bioceramics with tailored properties. This approach could prevent particle agglomeration and help maintain morphological stability, resulting in a uniform and porous structure. Another innovative aspect of obtaining HAp and 5ZnHAp powders through an adapted coprecipitation method (dried by lyophilization) lies in the ability of these methods to produce highly homogeneous, pure powders with controlled particle size and morphology. These powders could represent suitable candidates for uses in the biomedical field, offering a scalable, precise, and efficient approach to high-performance material synthesis. The resulting powders exhibit enhanced properties, making them ideal for biomedical applications such as bone regeneration and drug delivery, as well as industrial uses that demand precise material characteristics.

While optimizing and reproducing the process on a small scale is relatively straightforward, scaling up presents significant challenges. Ensuring reproducibility and maintaining the desired physicochemical and biological properties on a large scale are difficult, which limits the clinical and commercial applications of these nanometric biomaterials. Subtle variations in the manufacturing process can significantly impact critical properties such as size, crystallinity, and the loading/release profile of these ceramic biomaterials, ultimately affecting therapeutic outcomes. Producing these nanomaterials on a large scale necessitates well-equipped conventional pharmaceutical facilities to ensure that the handling capacity and safety of both the product and the environment are not compromised. The physicochemical properties and integrity of biomedical products derived from zinc ion-doped HAp powders must be preserved throughout the manufacturing process and their shelf life. Although many industries have established the infrastructure necessary for manufacturing various solid, liquid, and semi-solid nanomatrix medical products, the production of biomedical products at the nanoscale demands complex and costly techniques and technologies, such as high-energy grinding, synthesis under well-controlled conditions, high-pressure homogenization, and lyophilization. According to previous studies [110,111], a comprehensive understanding of the entire process, along with precise and regular verifications, standardized equipment, and validated processes and procedures, is essential. In addition, there is a need for highly qualified scientific and experimental personnel who can manage all the product quality parameters, such as physicochemical stability, size distribution, surface characteristics, loading, and porosity. In conclusion, we can say that understanding the fundamental, clinical, and regulatory aspects of nanoscale medicine is of paramount importance.

## 4. Conclusions

In this study, hydroxyapatite and zinc-doped hydroxyapatite powders were obtained through an adapted coprecipitation method and dried by lyophilization (HApLF and 5ZnHApLF). This study is the first to utilize lyophilization as a drying method for producing HAp and 5ZnHAp powders, resulting in materials with enhanced physicochemical and biological properties. More than that, the surface of the pellets was investigated by acoustic microscopy for the first time. The results of nondestructive ultrasound measurements provide valuable data about the stability of HApLF and 5ZnHApLF suspensions. The formation and presence of HAp in both studied samples was highlighted by the XRD and FTIR results. Valuable information about the purity of the HApLF and 5ZnHApLF chemical composition was provided by the results of the EDS and XPS studies. Our results reveal that the presence of Zn in the samples induces a slight shift of the FTIR maxima and some changes in the values of the lattice parameters. In addition, the presence of Zn ions in the HAp structure leads to a decrease in crystallite size. The from SEM, acoustic microscopy studies, and AFM studies showed that the roughness of HApLF is smaller than the 5ZnHApLF roughness, probably because the incorporation of zinc ions into the HAp matrix disrupts the crystal structure and determines variations in grain size, thus promoting a rougher surface morphology. The results of the cytotoxic assays highlighted that both HApLF and 5ZnHApLF exhibited excellent biocompatibility towards the MG63 and HGF-1 cell lines, showing a cell viability over 90%. Furthermore, additional information regarding the HGF-1 and MG63 cell development and proliferation on the surface of HApLF and 5ZnHApLF pellets was also presented using SEM and AFM. The results of the SEM observation highlighted that both MG63 and HGF-1 cells exhibited good adherence on the surface on HApLF and excellent adherence on 5ZnHApLF. The results demonstrated that MG63 cells adhered to the surfaces of both HApLF and 5ZnHApLF pellets and displayed a distinct flattened morphology with extensive cytoplasmic extensions. Furthermore, SEM images revealed enhanced cell spreading on the 5ZnHApLF surface, underscoring its superior biocompatibility and enhanced osteoconductive potential compared to the surface of the HApLF pellet. Furthermore, the AFM analysis revealed that MG63 and HGF-1 cells adhered to both HApLF and 5ZnHApLF surfaces and displayed a well-spread morphology with prominent extensions, reflecting strong interactions with the materials. In particular, the 5ZnHApLF surface exhibited an increased surface roughness and distinct topographical features that helped promote enhanced cellular adhesion and spreading, emphasizing its improved biocompatibility and osteoconductive potential compared to the HApLF surface. The results of the antimicrobial assays demonstrated that 5ZnHApLF possesses broad-spectrum antimicrobial activity against bacteria and fungi, with notable efficacy against *C. albicans*. These findings highlight the potential of 5ZnHApLF in applications such as antimicrobial coatings, biomedical implants, and infection-resistant materials.

## Figures and Tables

**Table 1:** Lattice parameters of HApLF and 5ZnHApLF.

Sample	a (Å)	c (Å)	c/a	V (Å3)	Xc	Crystallite Size (nm)
HAp (JCPDS no. 09–432)	9.418	6.884	0.73	528.80	-	-
HApLF	9.415	6.873	0.73	527.60	86.63	20.66
5ZnHApLF	9.427	6.864	0.728	528.25	86.03	16.92

**Table 2:** The surface atomic composition (atomic %).

Sample	C	O	Ca	P	Zn
HApLF	16.96	48.07	21.87	13.1	-
5ZnHApLF	14.6	52.3	20.2	12.4	0.5

**Table 3:** The value of (Ca + Zn)/P ratio obtained for HApLF and 5ZnHApLF.

Sample	Ratio Value
HApLF	Ca/P = 1.666
5ZnHApLF	(Ca + Zn)/P = 1.679

**Table 4:** Surface roughness parameters R_a_ and R_q_ (R_RMS_) of HApLF and 5ZnHApLF pellet surfaces obtained from SEM and AFM studies.

Sample	Characterization Method/Roughness Parameters			
	Scanning Electron Microscopy		Atomic Force Microscopy	
	R_q_ (nm)	R_a_ (nm)	R_q_ (nm)	R_a_ (nm)
HApLF	40.93	23.61	45.37	34.19
5ZnHApLF	79.48	51.49	96.61	80.03

**Table 5:** Hardness values obtained for HApLF and 5ZnHApLF pellets.

Sample	Hardness (GPa)
HApLF	3.1 ± 0.1
5ZnHApLF	3.5 ± 0.2

## Data Availability

The original contributions presented in this study are included in the article; further inquiries can be directed to the corresponding author.

## References

[1] Cowin S.C. (2001). Bone Mechanics Handbook.

[2] Scalera F., Gervaso F., Sanosh K.P., Sannino A., Licciulli A.J.C.I. (2013). Influence of the calcination temperature on morphological and mechanical properties of highly porous hydroxyapatite scaffolds. Ceram. Int..

[3] Wu Q., Zhang X., Wu B., Huang W. (2013). Effects of microwave sintering on the properties of porous hydroxyapatite scaffolds. Ceram. Int..

[4] Wang X., Ito A., Sogo Y., Li X., Oyane A. (2010). Zinc-containing apatite layers on external fixation rods promoting cell activity. Acta Biomater..

[5] Stanić V., Dimitrijević S., Antić-Stanković J., Mitrić M., Jokić B., Plećaš I.B., Raičević S. (2010). Synthesis, characterization and antimicrobial activity of copper and zinc-doped hydroxyapatite nanopowders. Appl. Surf. Sci..

[6] Al-esnawy A.A., Ereiba K.T., Bakr A.M., Abdraboh A.S. (2021). Characterization and antibacterial activity of Streptomycin Sulfate loaded Bioglass/Chitosan beads for bone tissue engineering. J. Mol. Struct..

[7] Khadtare S., Bansode A.S., Mathe V.L., Shrestha N.K., Bathula C., Han S.H., Pathan H.M. (2017). Effect of oxygen plasma treatment on performance of ZnO based dye sensitized solar cells. J. Alloys Compd..

[8] Sergi R., Bellucci D., Candidato R.T., Lusvarghi L., Bolelli G., Pawlowski L., Candiani G., Altomare L., De Nardo L., Cannillo V. (2018). Bioactive Zn-doped hydroxyapatite coatings and their antibacterial efficacy against *Escherichia coli* and *Staphylococcus aureus*. Surf. Coat. Technol..

[9] Fathi M.H., Hanifi A. (2007). Evaluation and characterization of nanostructure hydroxyapatite powder prepared by simple sol–gel method. Mater. Lett..

[10] Zhang X., Zhang Y., Ma G., Yang D., Nie J. (2015). The effect of the prefrozen process on properties of a chitosan/hydroxyapatite/poly (methyl methacrylate) composite prepared by freeze drying method used for bone tissue engineering. RSC Adv..

[11] Niziołek K., Słota D., Sadlik J., Łachut E., Florkiewicz W., Sobczak-Kupiec A. (2023). Influence of drying technique on physicochemical properties of synthetic hydroxyapatite and its potential use as a drug carrier. Materials.

[12] Alford N.M., Birchall J.D., Kendall K. (1986). Engineering ceramics—The process problem. Mater. Sci. Technol..

[13] Freedman M.R., Millard M.L. (1986). Improved Consolidation of Silicon Carbide. 10th Annual Conference on Composites and Advanced Ceramic Materials: Ceramic Engineering and Science Proceedings.

[14] Roosen A., Bowen H.K. (1988). Influence of various consolidation techniques on the green microstructure and sintering behavior of alumina powders. J. Am. Ceram. Soc..

[15] Predoi D., Iconaru S.L., Deniaud A., Chevallet M., Michaud-Soret I., Buton N., Prodan A.M. (2017). Textural, Structural and Biological Evaluation of Hydroxyapatite Doped with Zinc at Low Concentrations. Materials.

[16] Bouyer E., Gitzhofer F., Boulos M.I. (2000). Morphological study of hydroxyapatite nanocrystal suspension. J. Mater. Sci. Mater. Med..

[17] Hammond C. (2015). The Basics of Crystallography and Diffraction.

[18] Koutsopoulos S. (2002). Synthesis and characterization of hydroxyapatite crystals: A review study on the analytical methods. J. Biomed. Mater. Res..

[19] Landi E., Tampieri A., Celotti G., Sprio S. (2000). Densification behaviour and mechanisms of synthetic hydroxyapatites. J. Eur. Ceram. Soc..

[20] Predoi D., Iconaru S.L., Predoi M.V., Motelica-Heino M., Guegan R., Buton N. (2019). Evaluation of Antibacterial Activity of Zinc-Doped Hydroxyapatite Colloids and Dispersion Stability Using Ultrasounds. Nanomaterials.

[21] Iconaru S.L., Motelica-Heino M., Predoi D. (2013). Study on europium-doped hydroxyapatite nanoparticles by Fourier transform infrared spectroscopy and their antimicrobial properties. J. Spectrosc..

[22] Casa Software Ltd (2009). CasaXPS: Processing Software for XPS, AES, SIMS and More. www.casaxps.com.

[23] Biesinger M.C., Payne B.P., Grosvenor A.P., Lau L.W., Gerson A.R., Smart R.S.C. (2010). Resolving surface chemical states in XPS analysis of first row transition metals, oxides and hydroxides: Sc, Ti, V., Cu and Zn. Appl. Surf. Sci..

[24] Wagner C.D., Naumkin A.V., Kraut-Vass A., Allison J.W., Powell C.J., Rumble J.R. (2003). NIST Standard Reference Database 20, Version 3.4.

[25] Gwyddion. http://gwyddion.net/.

[26] Balakrishnan S., Padmanabhan V.P., Kulandaivelu R., Nellaiappan T.S.N., Sagadevan S., Paiman S., Mohammad F., Al-Lohedan H.A., Obulapuram P.K., Oh W.C. (2021). Influence of iron doping towards the physicochemical and biological characteristics of hydroxyapatite. Ceram. Int..

[27] Iconaru S.L., Predoi D., Ciobanu C.S., Motelica-Heino M., Guegan R., Bleotu C. (2022). Development of Silver Doped Hydroxyapatite Thin Films for Biomedical Applications. Coatings.

[28] (2013). Standard Test Method for Determining the Antimicrobial Activity of Antimicrobial Agents under Dynamic Contact Conditions.

[29] Iconaru S.L., Groza A., Gaiaschi S., Rokosz K., Raaen S., Ciobanu S.C., Chapon P., Predoi D. (2020). Antimicrobial Properties of Samarium Doped Hydroxyapatite Suspensions and Coatings. Coatings.

[30] LeGeros R.Z. (1991). Calcium Phosphates in Oral Biology and Medicine.

[31] Yu W., Sun T.W., Qi C., Ding Z., Zhao H., Zhao S., Shi Z., Zhu Y.J., Chen D., He Y. (2017). Evaluation of zinc-doped mesoporous hydroxyapatite microspheres for the construction of a novel biomimetic scaffold optimized for bone augmentation. Int. J. Nanomed..

[32] Miyaji F., Kono Y., Suyama Y. (2005). Formation and structure of zinc-substituted calcium hydroxyapatite. Mater. Res. Bull..

[33] Webster T.J., Ergun C., Doremus R.H., Bizios R. (2002). Hydroxylapatite with substituted magnesium, zinc, cadmium, and yttrium II: Mechanisms of osteoblast adhesion. J. Biomed. Mater. Res..

[34] Chen X., Tang Q.L., Zhu Y.J., Zhu C.L., Feng X.P. (2012). Synthesis and antibacterial property of zinc loaded hydroxyapatite nanorods. Mater. Lett..

[35] Hu W., Ma J., Wang J., Zhang S. (2012). Fine structure study on low concentration zinc substituted hydroxyapatite nanoparticles. Mater. Sci. Eng. C..

[36] Brook R.J. (1985). Processing technology for high performance ceramics. Mater. Sci Eng..

[37] Persson M., Forsgren A., Carlstrom E., Kall L., Kronberg B., Pompe R., Carlsson R., Vincenzini P. (1987). High Tech Ceramics.

[38] Fries R., Rand B., Cahn R.W., Hassen P., Kramer E.J. (1996). Materials Science and Technology, A Comprehensive Treatment in Processing of Ceramics Part I.

[39] Sahadat Hossain M., Ahmed S. (2023). FTIR spectrum analysis to predict the crystalline and amorphous phases of hydroxyapatite: A comparison of vibrational motion to reflection. RSC Adv..

[40] Bollino F., Armenia E., Tranquillo E. (2017). Zirconia/Hydroxyapatite Composites Synthesized Via Sol-Gel: Influence of Hydroxyapatite Content and Heating on Their Biological Properties. Materials.

[41] Predoi S.A., Ciobanu S.C., Chifiriuc C.M., Iconaru S.L., Predoi D., Negrila C.C., Marinas I.C., Raaen S., Rokosz K., Motelica-Heino M. (2024). Sodium bicarbonate-hydroxyapatite used for removal of lead ions from aqueous solution. Ceram. Int..

[42] Abifarin J.K., Obada D.O., Dauda E.T., Dodoo-Arhin D. (2019). Experimental data on the characterization of hydroxyapatite synthesized from biowastes. Data Brief.

[43] Singh A.V., Bansod G., Schumann A., Bierkandt F.S., Laux P., Nakhale S.V., Shelar A., Patil R., Luch A. (2024). Investigating Tattoo Pigments Composition with UV-Vis and FT-IR Spectroscopy supported by Chemometric Modelling. Curr. Anal. Chem..

[44] What is Adventitious Carbon?. http://www.xpsfitting.com/2011/01/what-is-adventitious-carbon.html.

[45] Davydov V., Rakhmanina A., Kireev I., Alieva I., Zhironkina O., Strelkova O., Dianova V., Samani T.D., Mireles K., Yahia L.H. (2014). Solid state synthesis of carbon-encapsulated iron carbide nanoparticles and their interaction with living cells. J. Mater. Chem. B.

[46] Roy A., Mukhopadhyay A.K., Das S.C., Bhattacharjee G., Majumdar A., Hippler R. (2019). Surface Stoichiometry and Optical Properties of Cux–TiyCz Thin Films Deposited by Magnetron Sputtering. Coatings.

[47] Rjeb M., Labzour A., Rjeb A., Sayouri S., El Idrissi M.C., Massey S., Adnot A., Roy D. (2004). Contribution to the study by x-ray photoelectron spectroscopy of the natural aging of the polypropylene. Moroc. J. Condens. Matter.

[48] Walker R.A., Wilson K., Lee A.F., Woodford J., Grassian V.H., Baltrusaitis J., Rubasinghege G., Cibin G., Dent A. (2012). Preservation of York Minster historic limestone by hydrophobic surface coatings. Sci. Rep..

[49] Negrila C.C., Predoi D., Ghita R.V., Iconaru S.L., Ciobanu S.C., Manea M., Badea M.L., Costescu A., Trusca R., Predoi G. (2021). Multi-Level Evaluation of UV Action upon Vitamin D Enhanced, Silver Doped Hydroxyapatite Thin Films Deposited on Titanium Substrate. Coatings.

[50] Wang F.H., Chang H.P., Tseng C.C., Huang C.C. (2011). Effects of H_2_ plasma treatment on properties of ZnO: Al thin films prepared by RF magnetron sputtering. Surf. Coat. Technol..

[51] Gu X., Zhang S., Zhao Y., Qiang Y. (2015). Band alignment of ZnO/ZnS heterojunction prepared through magnetron sputtering and measured by X-ray photoelectron spectroscopy. Vacuum.

[52] Singh A.V., Chandrasekar V., Prabhu V.M., Bhadra J., Laux P., Bhardwaj P., Al-Ansari A.A., Aboumarzouk O.M., Luch A., Dakua S.P. (2024). Sustainable bioinspired materials for regenerative medicine: Balancing toxicology, environmental impact, and ethical considerations. Biomed. Mater..

[53] Diez-Escudero A., Espanol M., Beats S., Ginebra M.P. (2017). In vitro degradation of calcium phosphates: Effect of multiscale porosity, textural properties and composition. Acta Biomater..

[54] Nathanael A.J., Mangalaraj D., Ponpandian N. (2010). Controlled growth and investigations on the morphology and mechanical properties of hydroxyapatite/titania nanocomposite thin films. Compos. Sci. Technol..

[55] Ahmed M.K., Al-Wafi R., Mansour S.F., El-Dek S.I., Uskoković V. (2020). Physical and biological changes associated with the doping of carbonated hydroxyapatite/polycaprolactone core-shell nanofibers dually, with rubidium and selenite. J. Mater. Res. Technol..

[56] Bhattacharjee P., Begam H., Chanda A., Nandi S.K. (2014). Animal trial on zinc doped hydroxyapatite: A case study. J. Asian Ceram. Soc..

[57] Poon C.Y., Bhushan B. (1995). Comparison of surface roughness measurements by stylus profiler, AFM and non-contact optical profiler. Wear.

[58] MacDonald W., Campbell P., Fisher J., Wennerberg A. (2004). Variation in surface texture measurements. J. Biomed. Mater. Res. B.

[59] Gadelmawla E.S., Koura M.M., Maksoud T.M., Elewa I.M., Soliman H.H. (2002). Roughness parameters. J. Mater. Process. Technol..

[60] Horváth R., Drégelyi-Kiss Á., Mátyási G. (2015). The Examination Of Surface Roughness Parameters In The Fine Turning Of Hypereutectic Aluminium Alloys. UPB Sci. Bull. Ser. D.

[61] Lebea L., Ngwangwa H.M., Desai D., Nemavhola F. (2021). Experimental investigation into the effect of surface roughness and mechanical properties of 3D-printed titanium Ti-64 ELI after heat treatment. Int. J. Mech. Mater. Eng..

[62] Wickramasinghe H.K. (1989). Scanned probe microscopes. Sci. Am..

[63] Toyoshima K., Yasumura M., Ohnishi N., Ueda Y. (1988). Quantitative evaluation of tablet sticking by surface roughness measurement. Int. J. Pharm..

[64] Rowe R.C. (1979). Surface roughness measurements on both uncoated and film-coated tablets. J. Pharm. Pharmacol..

[65] Radulescu D.-E., Vasile O.R., Andronescu E., Ficai A. (2023). Latest Research of Doped Hydroxyapatite for Bone Tissue Engineering. Int. J. Mol. Sci..

[66] Nisar A., Iqbal S., Atiq Ur Rehman M., Mahmood A., Younas M., Hussain S.Z., Tayyaba Q., Shah A. (2023). Study of physicomechanical and electrical properties of cerium doped hydroxyapatite for biomedical applications. Mater. Chem. Phys..

[67] Karunakaran G., Cho E.-B., Kumar G.S., Kolesnikov E., Govindaraj S.K., Mariyappan K., Boobalan S. (2023). CTAB enabled microwave-hydrothermal assisted mesoporous Zn-doped hydroxyapatite nanorods synthesis using bio-waste Nodipectennodosus scallop for biomedical implant applications. Environ. Res..

[68] Gu M., Li W., Jiang L., Li X. (2022). Recent progress of rare earth doped hydroxyapatite nanoparticles: Luminescence properties, synthesis and biomedical applications. Acta Biomater..

[69] Saxena V., Hasan A., Pandey L.M. (2018). Effect of Zn/ZnO integration with hydroxyapatite: A review. Mater. Technol..

[70] Ofudje E.A., Adeogun A.I., Idowu M.A., Kareem S.O. (2019). Synthesis and characterization of Zn-Doped hydroxyapatite: Scaffold application, antibacterial and bioactivity studies. Heliyon.

[71] Ahmed M.K., Ramadan R., Afifi M., Menazea A.A. (2020). Au-doped carbonated hydroxyapatite sputtered on alumina scaffolds via pulsed laser deposition for biomedical applications. J. Mater. Res. Technol..

[72] Li X., Yao C., Shen J., Zhu S., Kong Y., Yao C., Zhou Y., Xia J. (2024). The Impact of Titanium Hydroxyapatite Doping on the Mechanical and Biological Properties of Photocured Resin. Micromachines.

[73] Venkatasubbu G.D., Ramasamy S., Ramakrishnan V., Avadhani G.S., Thangavel R., Kumar J. (2011). Investigations on zinc doped nanocrystalline hydroxyapatite. Int. J. Nanosci. Nanotechnol..

[74] Uysal I., Severcan F., Tezcaner A., Evis Z. (2014). Co-doping of hydroxyapatite with zinc and fluoride improves mechanical and biological properties of hydroxyapatite. Prog. Nat. Sci. Mater. Int..

[75] Bensalem A., Kucukosman O.K., Raszkiewicz J., Topkaya F. (2021). Synthesis, characterization, bactericidal activity, and mechanical properties of hydroxyapatite nano powders impregnated with silver and zinc oxide nanoparticles (Ag-ZnO-Hap). Ceram. Int..

[76] Lu H., Qu Z., Zhou Y. (1998). Preparation and mechanical properties of dense polycrystalline hydroxyapatite through freeze-drying. J. Mater. Sci. Mater. Med..

[77] Tank K.P., Chudasama K.S., Thaker V.S., Joshi M.J. (2014). Pure and zinc doped nano-hydroxyapatite: Synthesis, characterization, antimicrobial and hemolytic studies. J. Cryst. Growth.

[78] Ren F., Xin R., Ge X., Leng Y. (2009). Characterization and structural analysis of zinc-substituted hydroxyapatites. Acta Biomater..

[79] Roohani N., Hurrell R., Kelishadi R., Schulin R. (2013). Zinc and its importance for human health: An integrative review. J. Res. Med. Sci..

[80] Dornelas J., Dornelas G., Rossi A., Piattelli A., Di Pietro N., Romasco T., Mourão C.F., Alves G.G. (2024). The Incorporation of Zinc into Hydroxyapatite and Its Influence on the Cellular Response to Biomaterials: A Systematic Review. J. Funct. Biomater..

[81] Thian E.S., Konishi T., Kawanobe Y., Lim P.N., Choong C., Ho B., Aizawa M. (2013). Zinc-substituted hydroxyapatite: A biomaterial with enhanced bioactivity and antibacterial properties. J. Mater. Sci. Mater. Med..

[82] Liu X., Xia Z., Wang Y., Luo D., Li Z., Meng Z., Lian H. (2024). Zinc-doped inorganic bioactive materials: A comprehensive review of properties and their applications in osteogenesis, antibacterial, and hemostasis. Appl. Mater. Today.

[83] Radovanović Ž., Veljović D., Jokić B., Dimitrijević S., Bogdanović G., Kojić V., Petrović R., Janaćković D. (2012). Biocompatibility and antimicrobial activity of zinc(II)-doped hydroxyapatite, synthesized by a hydrothermal method. J. Serb. Chem. Soc..

[84] Badea M.A., Balas M., Popa M., Borcan T., Bunea A.-C., Predoi D., Dinischiotu A. (2023). Biological Response of Human Gingival Fibroblasts to Zinc-Doped Hydroxyapatite Designed for Dental Applications—An In vitro Study. Materials.

[85] Buyuksungur S., Huri P.Y., Schmidt J., Pana I., Dinu M., Vitelaru C., Kiss A.E., Tamay D.G., Hasirci V., Vladescu A. (2023). In vitro cytotoxicity, corrosion and antibacterial efficiencies of Zn doped hydroxyapatite coated Ti based implant materials. Ceram. Int..

[86] He Z., Jiao C., Wu J., Gu J., Liang H., Shen L., Yang Y., Tian Z., Wang C., Jiang Q. (2023). Zn-doped chitosan/alginate multilayer coatings on porous hydroxyapatite scaffold with osteogenic and antibacterial properties. Int. J. Bioprint..

[87] Begam H., Kundu B., Chanda A., Nandi S.K. (2017). MG63 osteoblast cell response on Zn doped hydroxyapatite (HAp) with various surface features. Ceram. Int..

[88] Yamaguchi M., Oishi H., Suketa Y. (1987). Stimulatory effect of zinc on bone formation in tissue culture. Biochem. Pharmacol..

[89] Sogo Y., Ito A., Kamo M., Sakurai T., Onuma K., Ichinose N., Otsuka M., LeGeros R.Z. (2004). Hydrolysis and cytocompatibility of zinc-containing β-tricalcium phosphate powder. Mater. Sci. Eng. C.

[90] Boyan B.D., Hummert T.W., Dean D.D., Schwartz Z. (1996). Role of material surfaces in regulating bone and cartilage cell response. Biomaterials.

[91] Chung R.J., Hsieh M.F., Huang C.W., Perng L.H., Wen H.W., Chin T.S. (2006). Antimicrobial effects and human gingival biocompatibility of hydroxyapatite sol-gel coatings. J. Biomed. Mater. Res..

[92] Rotsch C., Radmacher M. (2000). Drug-induced changes of cytoskeletal structure and mechanics in fibroblasts: An atomic force microscopy study. Biophys. J..

[93] Murphy M.F., Lalor M.J., Manning F.C., Lilley F., Crosby S.R., Randall C., Burton D.R. (2006). Comparative study of the conditions required to image live human epithelial and fibroblast cells using atomic force microscopy. Microsc. Res. Technol..

[94] Gudur M.S.R., Rao R.R., Peterson A.W., Caldwell D.J., Stegemann J.P., Deng C.X. (2014). Noninvasive quantification of in vitro osteoblastic differentiation in 3D engineered tissue constructs using spectral ultrasound imaging. PLoS ONE.

[95] Deligianni D.D., Katsala N.D., Koutsoukos P.G., Missirlis Y.F. (2001). Effect of surface roughness of hydroxyapatite on human bone marrow cell adhesion, proliferation, differentiation and detachment strength. Biomaterials.

[96] Kim M.H., Kim B.S., Lee J., Cho D., Kwon O.H., Park W.H. (2017). Silk fibroin/hydroxyapatite composite hydrogel induced by gamma-ray irradiation for bone tissue engineering. Biomater. Res..

[97] Braet F., de Zanger R., Seynaeve C., Baekeland M., Wisse E. (2001). A comparative atomic force microscopy study on living skin fibroblasts and liver endothelial cells. Microscopy.

[98] Bushell G.R., Cahill C., Clarke F.M., Gibson C.T., Myhra S., Watson G.S. (1999). Imaging and force-distance analysis of human fibroblasts in vitro by atomic force microscopy. Cytom. J. Int. Soc. Anal. Cytol..

[99] Silva W.D.M., Ribeiro C.A., Marques C.S., Tabata A.S., Saeki M.J., Medeiros L.I., Oliveira D.E.D. (2017). Fibroblast and pre-osteoblast cell adhesive behavior on titanium alloy coated with diamond film. Mater. Res..

[100] Sprio S., Dapporto M., Preti L., Mazzoni E., Iaquinta M.R., Martini F., Tognon M., Pugno N.M., Restivo E., Visai L. (2020). Enhancement of the Biological and Mechanical Performances of Sintered Hydroxyapatite by Multiple Ions Doping. Front. Mater..

[101] Maleki-Ghaleh H., Siadati M.H., Fallah A., Koc B., Kavanlouei M., Khademi-Azandehi P., Moradpur-Tari E., Omidi Y., Barar J., Beygi-Khosrowshahi Y. (2021). Antibacterial and Cellular Behaviors of Novel Zinc-Doped Hydroxyapatite/Graphene Nanocomposite for Bone Tissue Engineering. Int. J. Mol. Sci..

[102] Dasgupta S., Banerjee S.S., Bandyopadhyay A., Bose S. (2010). Zn- and Mg-doped hydroxyapatite nanoparticles for controlled release of protein. Langmuir.

[103] Popa C.L., Deniaud A., Michaud-Soret I., Guégan R., Motelica-Heino M., Predoi D. (2016). Structural and Biological Assessment of Zinc Doped Hydroxyapatite Nanoparticles. J. Nanomater..

[104] Khan M.F., Hameedullah M., Ansari A.H., Ahmad E., Lohani M.B., Khan R.H., Alam M.M., Khan W., Husain F.M., Ahmad I. (2014). Flower-shaped ZnO nanoparticles synthesized by a novel approach at near-room temperatures with antibacterial and antifungal properties. Int. J. Nanomed..

[105] Phaechamuda T., Mahadlek J., Aroonrerk N., Choopun S., Charoenteeraboon J. (2012). Antimicrobial activity of ZnO-doxycycline hyclate thermosensitive gel. Sci. Asia.

[106] Peer D., Karp J.M., Hong S., Farokhzad O.C., Margalit R., Langer R. (2007). Nanocarriers as an emerging platform for cancer therapy. Nat. Nanotechnol..

[107] Anselmo A.C., Mitragotri S. (2016). Nanoparticles in the clinic. Bioeng. Transl. Med..

[108] Sainz V., Conniot J., Matos A.I., Peres C., Zupancic E., Moura L., Silva L.C., Florindo H.F., Gaspar R.S. (2015). Regulatory aspects on nanomedicines. Biochem. Biophys. Res. Commun..

[109] Kaur I.P., Kakkar V., Deol P.K., Yadav M., Singh M., Sharma I. (2014). Issues and concerns in nanotech product development and its commercialization. J. Control. Release.

[110] Desai N. (2012). Challenges in development of nanoparticle-based therapeutics. AAPS J..

[111] Ragelle H., Danhier F., Préat V., Langer R., Anderson D.G. (2017). Nanoparticle-based drug delivery systems: A commercial and regulatory outlook as the field matures. Expert Opin. Drug Deliv..

